# Novel Diagnostic and Therapeutic Options for *KMT2A*-Rearranged Acute Leukemias

**DOI:** 10.3389/fphar.2022.749472

**Published:** 2022-06-06

**Authors:** Bruno A. Lopes, Caroline Pires Poubel, Cristiane Esteves Teixeira, Aurélie Caye-Eude, Hélène Cavé, Claus Meyer, Rolf Marschalek, Mariana Boroni, Mariana Emerenciano

**Affiliations:** ^1^ Acute Leukemia RioSearch Group, Division of Clinical Research and Technological Development, Instituto Nacional de Câncer José Alencar Gomes da Silva (INCA), Rio de Janeiro, Brazil; ^2^ Bioinformatics and Computational Biology Laboratory, Instituto Nacional de Câncer José Alencar Gomes da Silva (INCA), Rio de Janeiro, Brazil; ^3^ Département de Génétique, UF de Génétique moléculaire, Assistance Publique des Hópitaux de Paris (AP-HP), Hópital Robert Debré, Paris, France; ^4^ INSERM UMR_S1131, Institut de Recherche Saint-Louis, Université de Paris-Cité, Paris, France; ^5^ DCAL/Institute of Pharmaceutical Biology, Goethe-University Frankfurt, Frankfurt am Main, Germany

**Keywords:** *KMT2A*, *MLL*, acute leukemia, biomarker, machine learning, therapy

## Abstract

The *KMT2A* (*MLL*) gene rearrangements (*KMT2A*-r) are associated with a diverse spectrum of acute leukemias. Although most *KMT2A*-r are restricted to nine partner genes, we have recently revealed that *KMT2A*-*USP2* fusions are often missed during FISH screening of these genetic alterations. Therefore, complementary methods are important for appropriate detection of any *KMT2A*-r. Here we use a machine learning model to unravel the most appropriate markers for prediction of *KMT2A*-r in various types of acute leukemia. A Random Forest and LightGBM classifier was trained to predict *KMT2A*-r in patients with acute leukemia. Our results revealed a set of 20 genes capable of accurately estimating *KMT2A*-r. The *SKIDA1* (AUC: 0.839; CI: 0.799–0.879) and *LAMP5* (AUC: 0.746; CI: 0.685–0.806) overexpression were the better markers associated with *KMT2A*-r compared to *CSPG4* (also named *NG2*; AUC: 0.722; CI: 0.659–0.784), regardless of the type of acute leukemia. Of importance, high expression levels of *LAMP5* estimated the occurrence of all *KMT2A-USP2* fusions. Also, we performed drug sensitivity analysis using IC50 data from 345 drugs available in the GDSC database to identify which ones could be used to treat *KMT2A*-r leukemia. We observed that *KMT2A*-r cell lines were more sensitive to 5-Fluorouracil (5FU), Gemcitabine (both antimetabolite chemotherapy drugs), WHI-P97 (JAK-3 inhibitor), Foretinib (MET/VEGFR inhibitor), SNX-2112 (Hsp90 inhibitor), AZD6482 (PI3Kβ inhibitor), KU-60019 (ATM kinase inhibitor), and Pevonedistat (NEDD8-activating enzyme (NAE) inhibitor). Moreover, IC50 data from analyses of *ex-vivo* drug sensitivity to small-molecule inhibitors reveals that Foretinib is a promising drug option for AML patients carrying *FLT3* activating mutations. Thus, we provide novel and accurate options for the diagnostic screening and therapy of *KMT2A*-r leukemia, regardless of leukemia subtype.

## Introduction

Chromosomal rearrangements involving the *KMT2A* (*MLL*) gene are recurrently associated with the disease phenotype of acute leukemia. Considering the high number of genes involved in *KMT2A* fusions (Meyer et al., 2018), the identification of *KMT2A* rearrangements (*KMT2A*-r) is routinely based on screening methods to guide the detection of this alteration regardless of the partner gene. Such methods include immunophenotyping using an antibody for detection of chondroitin sulfate proteoglycan 4 (CSPG4, also known as neuron-glial antigen 2, NG2) (Behm et al., 1996; Smith et al., 1996) or split-signal fluorescence *in situ* hybridization (FISH). Subsequently, the identification of the most frequent *KMT2A* fusion transcripts is generally performed by reverse transcription polymerase chain reaction (RT-PCR) (Burmeister et al., 2015), and long-distance inverse polymerase chain reaction (LDI-PCR) (Meyer et al., 2018). Recent studies have also demonstrated the application of next-generation sequencing (NGS) technologies for the detection of *KMT2A*-r, which also facilitates the identification of numerous genomic alterations for risk classification of acute leukemia (Shiba et al., 2019; Brown et al., 2020). However, the cost of NGS is still very high for most laboratories worldwide, and low-expression of *KMT2A* fusion transcripts provide limitations for precise detection of these alterations using RNA sequencing (RNA-Seq) in all *KMT2A*-r cases (Brown et al., 2020).

Recently, we have demonstrated that FISH fails to detect *KMT2A*-*USP2* fusions in most patients, leading to an underestimation of its frequency in acute leukemia (Meyer et al., 2019). Because this gene fusion derives from a short inversion within 11q23, 5′ and 3′ *KMT2A* probe signals usually do not split away from each other, mimicking a wild-type (normal) FISH pattern. The alteration is only observed whilst the inversion is accompanied by a 3′ *KMT2A* deletion. The challenge to accurately identify *KMT2A*-r is not restricted to FISH methodology. Several studies reveal a varying prevalence of false-negative results associated with NG2 detection by flow cytometry in patients with acute leukemia (Emerenciano et al., 2011; Menendez and Bueno, 2011). Therefore, a screening method that reduces the chances of false-negative results is still relevant in the context of *KMT2A*-r diagnosis.

In addition, the standard treatment offered for patients with *KMT2A*-r acute leukemia, which typically involves an intensive chemotherapy as induction, followed by additional consolidation therapy, has not yet significantly improved outcomes despite many years of various international efforts. Therefore, the urgent need for novel therapeutic strategies is unquestionable. Indeed, many studies are currently addressing new therapeutic options. MEK inhibitors have been pointed out as potential options for *KMT2A*-r patients carrying *RAS* mutations (Mansur et al., 2017; Kerstjens et al., 2018). The development of therapeutic strategies to specifically inhibit the recurrent fusion proteins may also be an opportunity, as previously reviewed (Steinhilber and Marschalek, 2018). Other promising options are based on the use of epigenetic drugs, such as demethylating agents and histone deacetylase inhibitors (Wong and So, 2020). Additionally, immunotherapy and the inhibition of PARP (Fritz et al., 2021) have shown potential to enhance the efficacy of other therapies and to ultimately overcome *KMT2A*-r acute leukemia. While the research timeline is running during the clinical testing of the aforementioned therapies, drug repurposing could meet the current need in a faster way (Tsakaneli and Williams, 2021).

In parallel, bioinformatics has been a branch of science which uses different methods and techniques like molecular biology, computer science, statistics, and artificial intelligence to design algorithms for solving biological problems. For instance, artificial intelligence encompasses a set of methodological approaches (e.g., machine learning) and has already been applied to understand cancer progression and to develop predictive models of diagnosis, prognosis and response to treatment in renal carcinoma and ovarian cancer (Jagga and Gupta, 2014; Xu et al., 2016). In this approach, the system can learn from varied data, predict and make decisions through classification and regression algorithms. The information used by the prediction model, whether molecular or genetic data, can be extracted, thus allowing the identification of new genes as possible important biomarkers for patient stratification and, consequently, for inferring better therapeutic strategies. Thus, machine learning approaches hold promise for a more robust and accurate characterization of hematologic malignancies (Eckardt et al., 2020). Furthermore, implementing machine learning methods will help clinicians analyze and interpret data, and increase objectivity, assisting clinical decision-making (Walter et al., 2021). Therefore, both the search for a therapeutic strategy and the search for a diagnostic biomarker can benefit from the availability of bioinformatics tools and machine learning approaches. Here, we evaluate the transcriptome of acute leukemia cases to point out therapeutic options, and to unravel appropriate markers for diagnostic routine prediction of *KMT2A*-r by integrating machine learning and bioinformatics approaches.

## Materials and Methods

### Patient Cohorts

The acute leukemia patients included in this study were selected based on known *KMT2A* status (*KMT2A-r* or *KMT2A* wild-type/*KMT2A*-WT) and available gene expression data from six different datasets (Supplementary Table S1). The Therapeutically Applicable Research to Generate Effective Treatments (TARGET) dataset (https://portal.gdc.cancer.gov/projects; dbGAP accession number phs000218) is composed of four different cohorts with pediatric patients diagnosed with 1) B-cell precursor acute lymphoblastic leukemia (B-ALL), 2) T-cell ALL (T-ALL), 3) acute myeloid leukemia (AML), and 4) acute leukemia of ambiguous lineage (ALAL). The diagnosis of *KMT2A*-r in TARGET dataset was obtained from the respective clinical data available in the TARGET Data Matrix (https://ocg.cancer.gov/programs/target/data-matrix; see the Common Data Element (CDE) files for more details). This identification was based on karyotype and FISH results for the B-ALL samples, FISH with confirmation by RNA-Seq (Liu et al., 2017) for the T-ALL cohort, RNA-Seq, Whole Genome Sequencing (WGS) and/or karyotype for the AML patients, and karyotype for the ALAL cohort. The Beat AML programme consists of a large dataset of AML pediatric and adult patients (data viewer: http://www.vizome.org/aml/). As previously described, the diagnosis of *KMT2A*-r in this cohort was obtained by conventional karyotype and RNA-Seq. All clinical data are found in the Supplementary Material of the original paper (Tyner et al., 2018).

The data from The European Genome-phenome Archive (EGA - https://ega-archive.org) study EGAS00001004212 (here called ALL-Ekert dataset) are from pediatric patients diagnosed with B-ALL and T-ALL. For this cohort, the *KMT2A* status was retrieved from the original study, which used conventional karyotype/FISH and RNA-Seq techniques (Brown et al., 2020). We also included data from adult patients diagnosed with AML deposited in the EGA database under the study accession number EGAS00001003096 (here called AML-Griffioen dataset). The identification of *KMT2A*-r was obtained by RNA-Seq analysis and validated by RT-PCR. The results were also previously published in the original paper (Arindrarto et al., 2021).

Another dataset evaluated in this work was composed of pediatric and adult AML samples from The Cancer Genome Atlas (TCGA) database (http://cancergenome.nih.gov/). To identify the patients with *KMT2A*-r, we used annotation of the structural variant from the “Acute Myeloid Leukemia” study in cBioPortal database (https://www.cbioportal.org/), which was based on RNA-Seq analysis (Gao et al., 2018). The last cohort included B-ALL, T-ALL and ALAL samples from a dataset of pediatric patients submitted to mRNA sequencing as part of the diagnostic workup at the Robert Debré hospital (Assistance Publique des Hôpitaux de Paris, AP-HP) in France (here called French dataset). The *KMT2A* status classification was based on both conventional cytogenetics and RNA-seq data.

### Gene Expression Data

All gene expression data were obtained from paired-end RNA-Seq methodology (Supplementary Table S1). For TARGET cohorts, we downloaded data corresponding to read counts (not normalized), when available, and FPKM (Fragments Per Kilobase Million) values of each gene annotated in the human reference genome GRCh37/hg19 (Ensembl release 59, Refseq and UCSC knownGene) from open-access files of these patients. The description of RNA-Seq data processing is available at the TARGET project portal (https://ocg.cancer.gov/programs/target/target-methods). In the case of ALAL samples, we used the read counts to calculate FPKM values using the Bioconductor R package “edgeR” version 3.30.3 (Robinson et al., 2010; McCarthy et al., 2012). For the Beat AML dataset, we downloaded gene expression log_2_ normalized FPKM values for each sample from the Supplementary Table S8 in the original paper (Tyner et al., 2018). We converted the log_2_ FPKM values to normal FPKM values by raising 2 to the power of the logarithmic values so that they would be correctly used in further analysis. Gene assignments were also based on the human reference genome GRCh37 (Ensembl release 75). For more details on processing steps, please see the methods section in the reference (Tyner et al., 2018). The gene expression data of the TCGA dataset were downloaded using the Bioconductor R package “TCGAbiolinks” version 2.16.4 (Colaprico et al., 2016). Through the GDCquery function, we used the following parameters to obtain the FPKM values of data aligned to the human reference genome GRCh38 (UCSC knownGene) (Gao et al., 2019): project = “TCGA-LAML,” data.category = “Transcriptome Profiling,” data.type = “Gene expression Quantification,” workflow.type = “HTSeq - FPKM,” legacy = False.

The fastq files from ALL-Ekert and AML-Griffioen datasets were downloaded *via* the EGA download client, pyEGA3 (https://github.com/EGA-archive/ega-download-client), after gaining access permission to EGA studies EGAS00001004212 and EGAS00001003096, respectively. The reads were trimmed using the software Trimmomatic version 0.39 (Bolger et al., 2014) and then were mapped to the human reference genome GRCh37 (Ensembl release 82) using STAR algorithm version 2.6.0c (Dobin et al., 2013). The RSEM software package version 1.3.0 (Li and Dewey, 2011) was used to estimate read counts and FPKM values of each gene annotated.

The sequencing and gene expression data generation of all samples included in the French dataset were performed by IntegraGen. Libraries were prepared with the NEBNextUltra II Directional RNA Library Prep Kit for Illumina protocol, according to supplier recommendations, and sequencing was carried out on Illumina NovaSeq (paired-end 100 bp reads). The STAR alignment algorithm (Dobin et al., 2013) was used to obtain the number of reads associated to each gene in the human reference genome GRCh37 by the Gencode release 31 annotation (restricted to protein-coding genes, antisense and long intervening noncoding RNAs). Raw counts for each sample were imported into R statistical software. Extracted count matrix was normalized for library size and coding length of genes to compute FPKM expression levels.

We also included RNA-seq data from human acute leukemia cell lines for validation analysis. The gene expression data, cell lines annotations and *KMT2A* fusion information were retrieved from the Cancer Cell Line Encyclopedia (CCLE) database (https://sites.broadinstitute.org/ccle/) at the Dependency Map (DepMap) portal (https://depmap.org/portal/download/; CCLE 2019 release). RNA-seq profiling was performed as previously described (Ghandi et al., 2019), and reads were aligned to the GRCh37 human genome reference. The gene expression was quantified in the TPM (Transcripts Per Million) unit.

### Machine Learning Analysis

The workflow used in this step is described in Supplementary Figure S1. For the identification of predictive markers of *KMT2A*-r using machine learning approaches, we obtained a unique gene expression data with the FPKM normalized values of 14,287 genes. Ensembl IDs were converted to gene IDs using the Bioconductor R package “biomaRt” version 2.44.4 (Durinck et al., 2005; Durinck et al., 2009), and duplicate genes were removed. We also added a batch effect correction step in order to remove the variability from the different datasets using the removeBatchEffect function of the Bioconductor R package “limma” version 3.44.3 (Ritchie et al., 2015). Then, the continuous variables were standardized using z-score. Variables with a correlation greater than 0.90 or low variance (zero or near zero) were excluded. Then, a wrapper feature selection step was performed to reduce the dimensionality of this dataset using BorutaPy version 0.3 (Kursa and Rudnicki, 2010). The acute leukemia subtype and age group information—pediatric (<22 years-old), younger adults (≥22 years-old and <60 years-old), and older adults (≥60 years-old)—were also evaluated for the construction of the model. Variables with more than two categories were represented by a set of dummy variables, with one variable for each category. The Random Forest and LightGBM models were trained with 70% of the data (training set), and tested in the remaining 30% (testing set). The 30-folds cross validation was used to adjust hyperparameters with the GridSearchCV function. Due to class imbalance, we resampled our training dataset using the SMOTEENN method, which is an interesting technique that combines both undersampling [using Edited Nearest Neighbor (ENN)] and oversampling (SMOTE).

Afterwards, accuracy, sensitivity, specificity, positive predictive value (PPV) and negative predictive value (NPV) were analyzed to assess the performance of our model. The combination of these parameters with the value of the area under the receiver operating characteristic (ROC) curve (AUC) was used to select the best model. Besides that, we calculated the Shapley values of each feature according to the predictive model to understand their respective contributions using SHAP (SHapley Additive exPlanations) version 0.36.0 (Lundberg et al., 2017). All previous analyzes were performed using the Python programming language with the scikit-learn library (Pedregosa et al., 2011). Heatmaps were constructed with the CRAN R package “pheatmap” version 1.0.12 (Kolde, 2019) using log_2_ (FPKM + 1) values, z-scaled across samples. Genes (rows) were clustered using Pearson correlation, and samples (columns) were clustered using Euclidean distance. This step was performed in the R statistical environment version 4.0.5.

### Pathway Enrichment Analysis

The gene list obtained from machine learning step was evaluated for known functional processes by the Kyoto Encyclopedia of Genes and Genomes (KEGG) database using the over-representation analysis (ORA) methodology in the WEB-based GEne SeT AnaLysis Toolkit (Liao et al., 2019)*.* We used genome protein-coding as a reference Set. *p* values were controlled for false discovery rate (FDR) using the Benjamini–Hochberg method, and we considered FDR ≤ 0.05 as significant results.

### Selection of Candidate Drugs for *KMT2A*-r

The half-maximal inhibitory concentration (IC50) z-score values of the drugs with data available in the Genomics of Drug Sensitivity in Cancer (GDSC) repository (Yang et al., 2013) were downloaded for acute leukemia cell lines from GDSC1 dataset (https://www.cancerrxgene.org/downloads/bulk_download). We also downloaded IC50 values of some small-molecule inhibitors from the Supplementary Table S8 of Beat AML dataset (Tyner et al., 2018). The data were obtained from *ex vivo* functional drug screening analyses using freshly isolated mononuclear cells from AML samples.

To search for drug-gene interactions, for which *KMT2A*-r cell lines demonstrated sensitivity was used as an input in the web resource Drug-Gene Interaction database (DGIdb; https://www.dgidb.org/search_interactions) (Freshour et al., 2021). The results generated by this tool were downloaded as a tab-separated values (TSV) file.

### Statistical Analysis

The ROC curves and AUC values were generated by the CRAN R package “pROC” version 1.17.0.1 (Robin et al., 2011). Association analyses were represented by box plots using the CRAN R package “ggplot2” version 3.3.3 (Wickham, 2016). Expression and IC50 data were compared between two or more groups with the unpaired two-samples Wilcoxon test and the Kruskal-Wallis test, respectively. *p*-values < 0.05 were considered statistically significant. All analyses presented in this study, with exception of the machine learning step, were performed in the R statistical environment version 4.0.5.

## Results

### Dataset Description

A total of 1,659 acute leukemia samples were evaluated in this study, consisting of 899 AML, 415 B-ALL, 273 T-ALL and 72 ALAL from six different datasets. These samples were mostly obtained at the time of diagnosis (90.3%) and refer to pediatric patients (61.4%) (Table 1). The frequency of cytogenetic/molecular alterations in each acute leukemia subtype is shown in Supplementary Figure S2. We included 121 *KMT2A*-r cases (7.3%) and classified the other molecular subgroups as *KMT2A*-WT (*n* = 1,538; 92.7%). As expected, *KMT2A*-r were more frequently in the pediatric age group, and less frequently in the older adults, when compared to *KMT2A*-WT (76.0% vs. 60.3%, and 5.0% vs. 20.4%, respectively; *p* = 0.0001).

**TABLE 1:** Cohort characterization.

Variables	Overall		Acute leukemias								*KMT2A* status				
			B-ALL		T-ALL		AML		ALAL		*KMT2A*-r		*KMT2A*-WT		*p*-value^a^
Age group^b^															**0.0001**
Pediatric	1019	(61.4)	414	(99.8)	270	(98.9)	263	(29.3)	72	(100.0)	92	(76.0)	927	(60.3)	
Younger adult	321	(19.3)	1	(0.2)	3	(1.1)	317	(35.3)	0	(0.0)	23	(19.0)	298	(19.4)	
Older adult	319	(19.2)	0	(0.0)	0	(0.0)	319	(35.5)	0	(0.0)	6	(5.0)	313	(20.4)	
Sex															0.1388
Female	696	(42.0)	190	(45.8)	78	(28.6)	399	(44.4)	29	(40.3)	59	(48.8)	637	(41.4)	
Male	963	(58.0)	225	(54.2)	195	(71.4)	500	(55.6)	43	(59.7)	62	(51.2)	901	(58.6)	
Sample type															—
Diagnosis	1498	(90.3)	395	(95.2)	271	(99.3)	760	(84.5)	72	(100.0)	118	(97.5)	1380	(89.7)	
Refractory	93	(5.6)	0	(0.0)	0	(0.0)	93	(10.3)	0	(0.0)	1	(0.8)	92	(6.0)	
Relapse	45	(2.7)	20	(4.8)	2	(0.7)	23	(2.6)	0	(0.0)	2	(1.7)	43	(2.8)	
Remission	18	(1.1)	0	(0.0)	0	(0.0)	18	(2.0)	0	(0.0)	0	(0.0)	18	(1.2)	
Unknown	5	(0.3)	0	(0.0)	0	(0.0)	5	(0.6)	0	(0.0)	0	(0.0)	5	(0.3)	
*KMT2A* status															—
*KMT2A*-r	121	(7.3)	22	(5.3)	14	(5.1)	77	(8.6)	8	(11.1)	—		—		
*KMT2A*-WT	1538	(92.7)	393	(94.7)	259	(94.9)	822	(91.4)	64	(88.9)	—		—		
Total	1659	(100.0)	415	(100.0)	273	(100.0)	899	(100.0)	72	(100.0)	121	(100.0)	1538	(100.0)	

a Pearson’s Chi-squared test.

b Pediatric (<22 years); Adult—younger adults (≥22 years and <60 years) and older adults (≥60 years).

bold value, p < 0.05.

### A Machine Learning Model for *KMT2A*-r Prediction

For the algorithm development, we selected the data from four of the six datasets: TARGET, Beat AML, ALL-Ekert, and AML-Griffioen. We used gene expression data of 14,287 genes from 1,332 acute leukemia samples, including 100 *KMT2A*-r and 1,232 *KMT2A*-WT cases. First, we performed a feature selection analysis to reduce the dimensionality of the dataset. As a result, 247 genes were selected due to their potential to predict *KMT2A*-r (Supplementary File S1), such as *CSPG4*, *MLLT10*, *MEIS1* and several genes of *HOX* family (*HOXA3*, *HOXA4*, *HOXA5*, *HOXA6*, *HOXA7*, *HOXA9* and *HOXA10*). Among them, 176 genes had increased expression in *KMT2A*-r acute leukemias (Supplementary Figure S3A). The enrichment analysis revealed a significant association of 13 genes (*ETV1*, *FCGR1A*, *HOXA10 HOXA9*, *MEF2C*, *MEIS1*, *PBX3*, *PROM1*, *RUNX2*, *SMAD1*, *SPINT1*, *SUPT3H* and *ZEB1*) with the “Transcriptional misregulation in cancer” pathway (FDR < 0.001; Supplementary Figure S3B). It is noteworthy that, based on KEGG Gene Set (hsa05202), *HOXA9*, *HOXA10* and *MEIS1* have association with differentiation resistance in *KMT2A*-*MLLT1* (or *MLL*-*ENL*) T-ALL, while this same biological process has the involvement of *PBX3*, *RUNX2*, *SMAD1*, *MEF2C*, *HOXA9* and *HOXA10* in *KMT2A*-*AFF1* (or *MLL*-*AF4*) B-ALL. The *SUPT3H* gene is a chromatin regulator and *PROM1* is a signaling mediator, both are also associated with *KMT2A*-*AFF1* (or *MLL*-*AF4*) in B-ALL.

**FIGURE 1:**
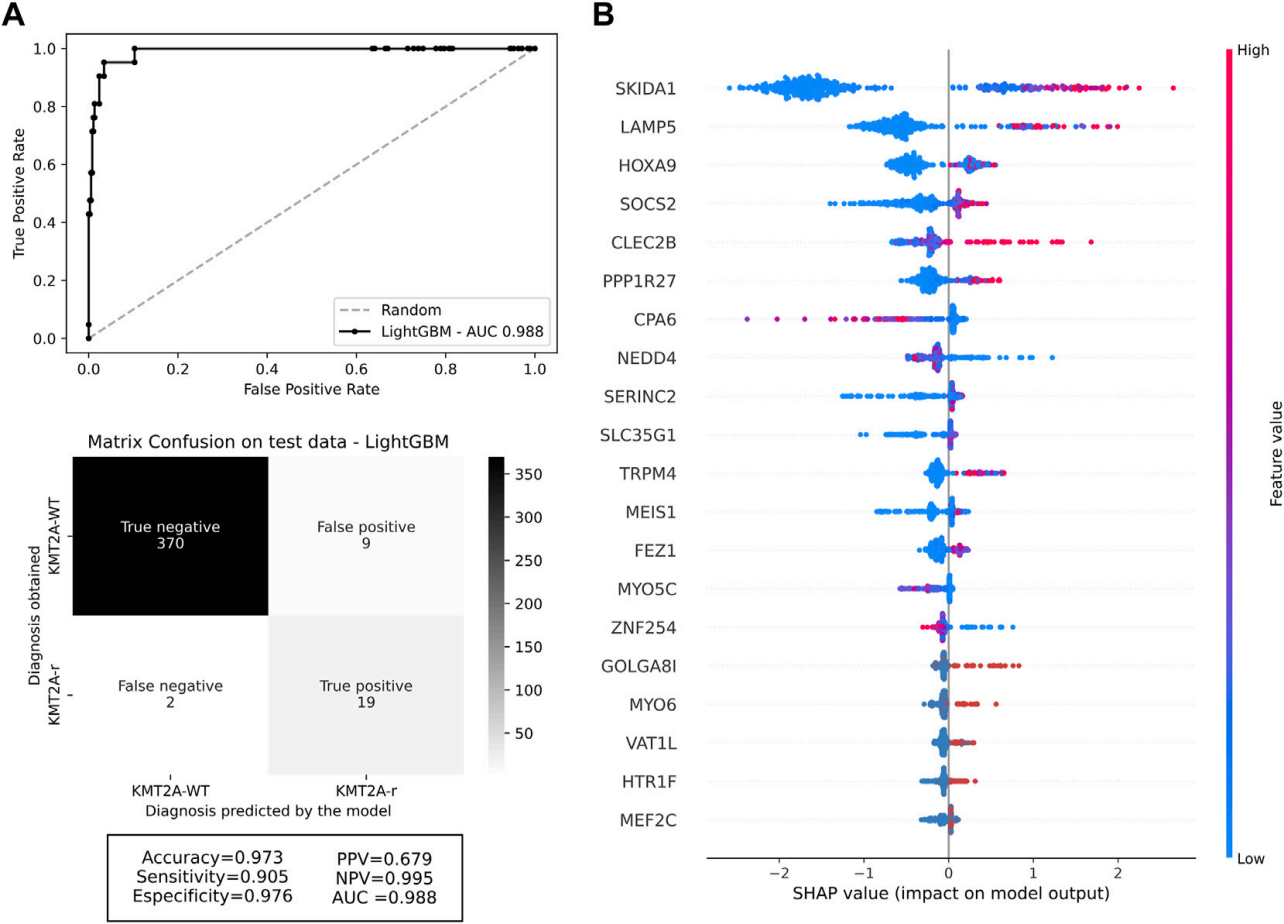
Machine learning performance. **(A)** ROC curve of the selected machine learning model with 247 genes, and the confusion matrix with performance measures of testing data. The darker the quadrant color, the greater the number of patients. Respective performance measures are below the matrix. **(B)** Feature contributions according to the shapley values. On the *x*-axis, positive and negative values are correlated with prediction of *KMT2A*-r and *KMT2A*-WT, respectively. Each dot represents one patient, colored according to the feature value (red fading to blue means high to low values).

With a final dataset composed by 247 genes and 1,332 samples, we randomly split our cohort into 70% training (932 samples, 79 *KMT2A*-r and 853 *KMT2A*-WT) and 30% testing (400 samples, 21 *KMT2A*-r and 379 *KMT2A*-WT) data subsets (Supplementary Tables S2, S3). First, we used the Random Forest and LightGBM algorithms to build a better model based on the 247 selected features and some well-known features associated with *KMT2A*-r, such as age group and acute leukemia subtypes. After the *KMT2A*-r prediction in the testing dataset, we compared the performance measures of each model. In general, we had similar performances between the different models (Supplementary Table S4a). However, we chose the model built with the LightGBM algorithm and 247 genes without clinical variables due to the best combination of AUC, accuracy, sensitivity and specificity (0.988, 0.973, 0.905, 0.976, respectively). In other words, given the testing dataset, our model was able to predict 19 of 21 *KMT2A*-r patients and 370 of 379 *KMT2A*-WT patients (Figure 1A). In order to reduce the gene set list, we selected the top 20 genes with the greatest contributions to the prediction of *KMT2A*-r in the LightGBM algorithm model, as indicated by the Shapley values (Figure 1B). Most genes are upregulated in *KMT2A*-r patients in comparison to *KMT2A*-WT patients (Supplementary Figure S3C). In contrast, *CPA6*, *NEDD4*, *ZNF254* and *MYO5C* had reduced expression in *KMT2A*-r cases. We also validated the expression of these genes in human acute leukemia cell lines with and without *KMT2A*-r. Our results showed the same expression profile observed in acute leukemia patients, given the clusterization of *KMT2A*-r cell lines (exception of RS4; 11 B-ALL cell line) (Supplementary Figure S4). Together, these data suggest that our model selected a set of genes highly correlated with *KMT2A*-r.

**FIGURE 2:**
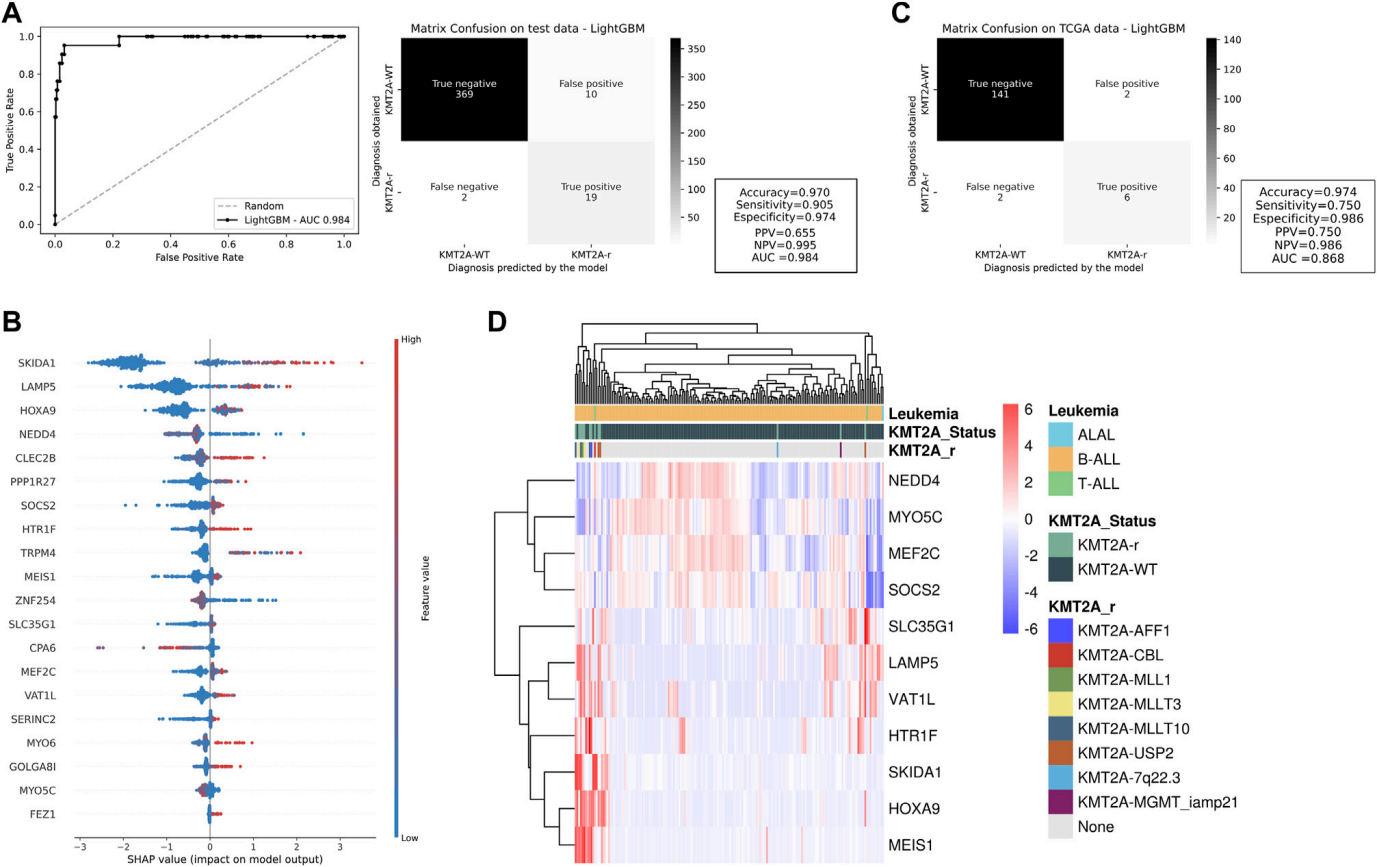
Testing and validation of potential *KMT2A*-r predictors. **(A)** ROC curve and confusion matrix of the test cohort for the machine learning model with top 20 genes. The darker the quadrant color, the greater the number of patients. The machine learning performance measures are beside the matrix. **(B)** Feature contributions of each gene included in this model according to the shapley values. On the *x*-axis, positive and negative values are correlated with prediction of *KMT2A*-r and *KMT2A*-WT, respectively. Each dot represents one patient, colored according to the feature value (red fading to blue means high to low values). **(C)** The performance measures of our model in an independent cohort (TCGA dataset including AML patients), represented by the confusion matrix, and a **(D)** heatmap illustrating the expression of 11 *KMT2A*-r predictor genes as indicated by the machine learning analysis. The clusterization was performed in the French dataset with ALL patients.

With training and testing data subsets now composed of 20 genes, we used LightGBM algorithms again to build four additional different models also based on selected features and clinical variables associated with *KMT2A*-r. The performance measures of these models can be accessed in Supplementary Table S4b. We chose the model trained and tested with the 20 genes and acute leukemia subtype variables as our final prediction model for *KMT2A*-r, with AUC of 0.984, accuracy of 0.970, sensitivity of 0.905, and specificity of 0.974 (Figure 2A). It is noteworthy that, even with a significant reduction of features, our final model was able to predict the *KMT2A*-r cases with a good performance (19 of 21 *KMT2A*-r and 369 of 379 *KMT2A*-WT cases). We observed some feature modifications in Shapley values (Figure 2B), however, the two most relevant genes to the model (*SKIDA1* and *LAMP5*) did not change.

To validate our *KMT2A*-r prediction model, we evaluated its performance in an independent subset. The TCGA cohort included 151 AML patients classified according to *KMT2A* status: *KMT2A*-WT (*n* = 143) and *KMT2A*-r (*n* = 8). The *KMT2A* partner genes in these patients were: *MLLT10* (*n* = 1), *ELL* (*n* = 3), *MLLT3/AF9* (*n* = 2), and *MLLT4* (*n* = 2). With a whole new set of data, our model was able to correctly predict six of eight *KMT2A*-r samples and 141 of 143 *KMT2A*-WT cases. Therefore, we created a good predictor for *KMT2A*-r samples (AUC = 0.868), with higher accuracy, sensitivity, and specificity (0.974, 0.750, and 0.986, respectively; Figure 2C).

We also analyzed one French dataset, which included 176 pediatric patients with acute leukemia (173 B-ALL, 2 T-ALL, and 1 ALAL), consisting of 163 *KMT2A*-WT and 13 *KMT2A*-r. Of notice, *KMT2A* in-frame fusions were associated with *USP2* (*n* = 3), *MLLT10* (*n* = 3), *MLLT3/AF9* (*n* = 3), *AFF1/AF4* (*n* = 1), and *CBL* (*n* = 1). Two patients had out-of-frame fusions with *MGMT* (*n* = 1) or within the 7q22.3 region (*n* = 1). Therefore, we evaluated whether the other genes (11 of 20 genes) would still be able to cluster *KMT2A*-r patients. As expected, the two samples with out-of-frame did not present a similar expression profile of the other *KMT2A* fusions, as well as one sample with *KMT2A-USP2*, which had higher expression of *NEDD4* and lower expression of *SKIDA1*, *HOXA9* and *MEIS1* (Figure 2D). On the other hand, we demonstrated that our panel of genes is highly associated with the *KMT2A*-r phenotype. The information regarding false negative and false positive groups were also described (Supplementary Table S5). We observed that *KMT2A*-r cases wrongly predicted by our model consist of rare fusions, which were under or not represented in the training dataset.

### Biomarkers for Prediction of *KMT2A*-r in Acute Leukemia

In order to find an optimal biomarker for *KMT2A*-r identification, we next evaluated the two most important genes ranked by the selected machine learning model (*SKIDA1* and *LAMP5*; Figures 1B, 2B) in comparison with the *CSPG4* gene, which encodes NG2. We evaluated their expression by cytogenetic/molecular subgroups of AML, B-ALL, T-ALL, and ALAL (Supplementary Figure S5). Although all genes presented variable expression in individual subgroups of acute leukemia, *SKIDA1* had pronounced expression in *KMT2A*-r compared to the remaining subgroups in all acute leukemia subtypes. *SKIDA1* was significantly overexpressed in all molecular subgroups, except for only two subgroups of AML (*DEK-NUP214*, and other alterations). *LAMP5* expression was also not different in *DEK-NUP214* and complex karyotype samples when compared to *KMT2A*-r in AML, as well as hypodiploid cases in B-ALL. Besides that, *LAMP5* does not appear to be a good biomarker for *KMT2A*-r in T-ALL. Similarly, *CSPG4* expression was comparable between *KMT2A*-r and all other subgroups of T-ALL. On the other hand, *SKIDA1* had the highest expression in *KMT2A*-r as compared to all remaining subgroups of T-ALL (*p* < 0.01).

**FIGURE 3:**
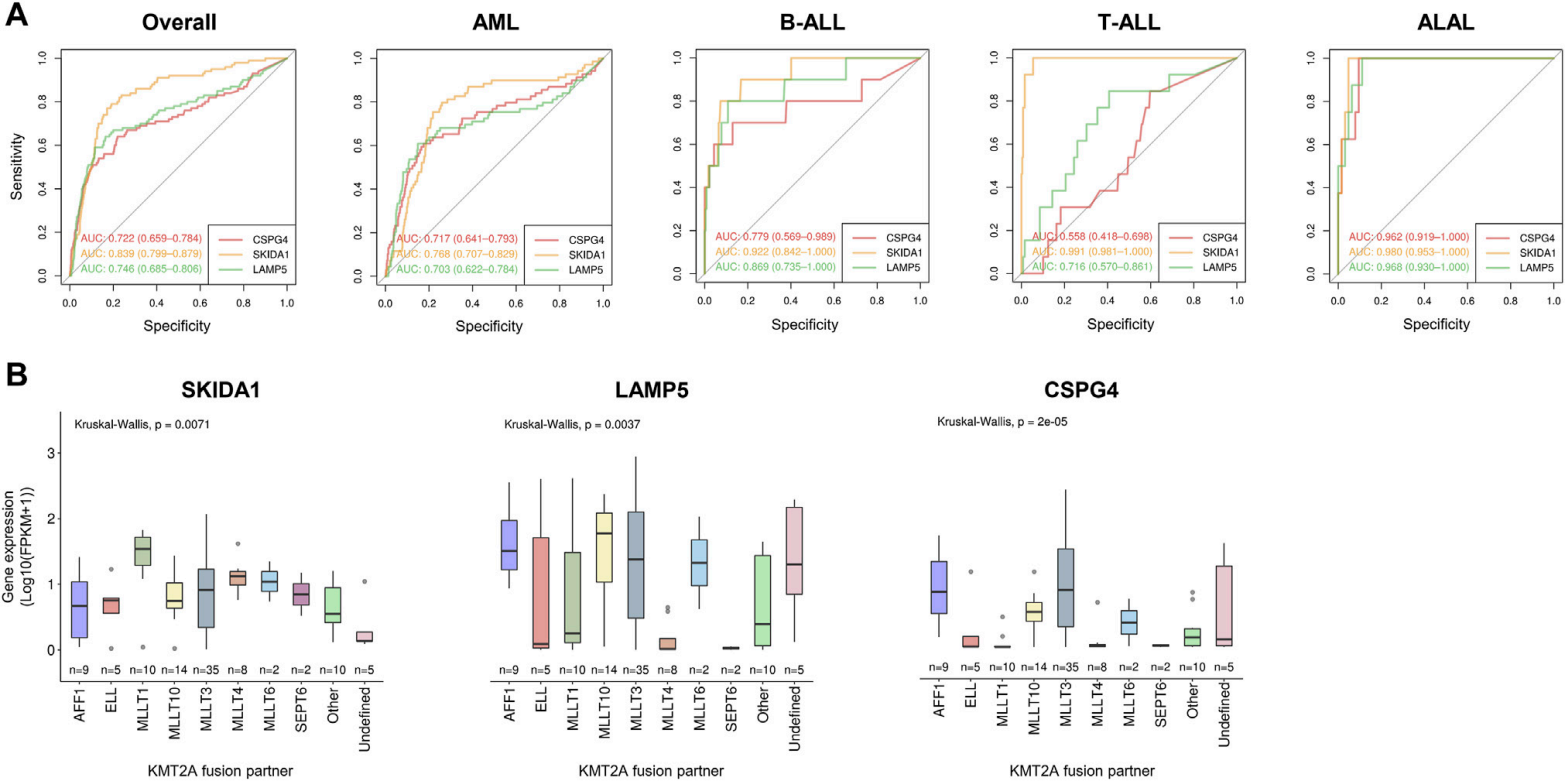
Relationship between *SKIDA1*, *LAMP5,* and *CSPG4* expression and *KMT2A*-r in acute leukemia. **(A)** ROC curves and AUC values of each gene transcript according to acute leukemia subtypes. **(B)** Transcript expression among varied *KMT2A* fusions.

To evaluate the performance of these genes on *KMT2A*-r classification, we plotted ROC curves and calculated their respective AUC values among acute leukemia subtypes (Figure 3A). In general, *SKIDA1* (AUC: 0.839; CI: 0.799–0.879) and *LAMP5* (AUC: 0.746; CI: 0.685–0.806) performed better than *CSPG4* (AUC: 0.722; CI: 0.659–0.784). Considering each subtype of leukemia, the most divergent performance was observed in T-ALL, in which *SKIDA1* had the best estimates (AUC: 0.991; CI: 0.981–1.000), followed by *LAMP5* (AUC: 0.716; CI: 0.570–0.861), and *CSPG4* (AUC: 0.558; CI: 0.418–0.698). The most similar results were associated with ALAL, in which those three genes provided optimal estimates of *KMT2A*-r. Next, we obtained the best thresholds indicated by ROC curves for *SKIDA1*, *LAMP5* and *CSPG4* in acute leukemia (1.928, 2.874, and 0.401, respectively), AML (2.303, 6.002, and 1.371, respectively), and B-ALL (0.227, 5.846, and 0.305, respectively) to validate the predictive power of these genes as biomarkers in the TCGA (151 AML patients) and the French (173 B-ALL patients) cohorts. These results were demonstrated through confusion matrices (Supplementary Figure S6) and performance metrics (Supplementary Table S6). Overall, the expression of either *SKIDA1* or *LAMP5* was more accurate for estimating *KMT2A*-r as compared with *CSPG4* expression. In our validation analyses, *SKIDA1* was associated with a higher sensitivity (0.875 vs. *CSPG4* = 0.625) and specificity (0.734 vs. *CSPG4* = 0.573) in AML, while *LAMP5* had greater performance in B-ALL (sensitivity = 0.833 vs. *CSPG4* = 0.417; specificity = 0.820 vs. *CSPG4* = 0.870) if the overall acute leukemia threshold was used. Similar results were also demonstrated when thresholds for the respective acute leukemia subtype (AML or B-ALL) were used. It is important to highlight that the validation cohort contains three B-ALL patients with *KMT2A-USP2*. Of note, *LAMP5* was extremely overexpressed in these patients (the median FPKM was 122.13, 0.15, 33.25, for *KMT2A-USP2*, *KMT2A*-WT, and other molecular subgroups, respectively), thus allowing the prediction of this gene fusion in all cases. On the other hand, *CSPG4* failed to predict it in one of these cases, and *SKIDA1* was not able to predict this rearrangement in all cases either.

Because *KMT2A* is a promiscuous gene and the distribution of fusion partners varies according to leukemia subtypes, we hypothesized that *SKIDA1* and *LAMP5* expression could be associated with specific gene rearrangements (Supplementary Table S7). The expression of *SKIDA1*, *LAMP5*, and *CSPG4* varied according to each *KMT2A*-r, but the latter had the most remarkable variation levels (*p* = 0.00002). Notably, *CSPG4* expression was lower in *KMT2A* fusions with *ELL*, *MLLT1*, *MLLT4*, and *SEPT6*. Conversely, *SKIDA1* had the lowest expression variation across *KMT2A* fusions (Figure 3B).

### Therapeutic Options for *KMT2A*-r Acute Leukemia

**FIGURE 4:**
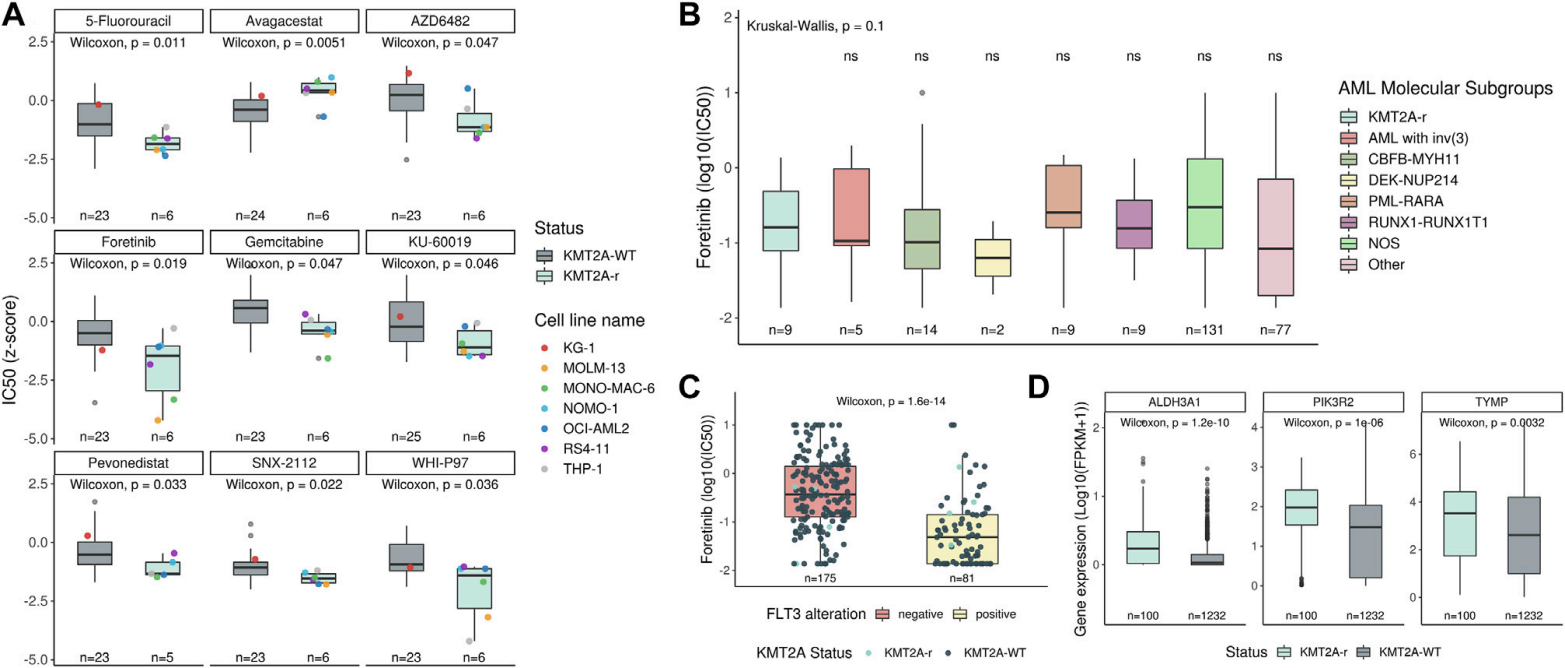
Identification of therapeutic drugs for *KMT2A*-r leukemia. **(A)** Comparison of the IC50 data, available in the GDSC database, to several drugs between *KMT2A*-WT and *KMT2A*-r leukemia cell lines. The KG-1 (red point) is a human AML cell line, with a variant, KG-1a, known to be resistant to chemotherapy. Comparison of the sensitivity to foretinib between **(B)** AML molecular subgroups and *KMT2A*-r, as well as **(C)** *FLT3* wild-type (red boxplot) and *FLT3* mutated (yellow boxplot). **(D)** Transcript expression compared between *KMT2A*-WT and *KMT2A*-r in acute leukemia samples included in this study.

Since *KMT2A*-r are often associated with chemo-refractory acute leukemia, several studies aimed to search for new possibilities of either new targets or drugs for the treatment of this leukemia subtype. Here, we evaluated drug screening for 33 acute leukemia cell lines (20 AML and 13 ALL) using the IC50s of 345 drugs available in the GDSC database (Supplementary Table S8). After comparing *KMT2A*-r (*n* = 6) with *KMT2A*-WT (*n* = 27), we observed that *KMT2A*-r cell lines were more sensitive to 5-Fluorouracil (5FU) and Gemcitabine (both antimetabolite chemotherapy drugs; *p* = 0.01 and *p* = 0.04, respectively), WHI-P97 (JAK-3 inhibitor; *p* = 0.03), Foretinib (MET/VEGFR inhibitor; *p* = 0.01), SNX-2112 (Hsp90 inhibitor; *p* = 0.02), AZD6482 (PI3Kβ inhibitor; *p* = 0.04), KU-60019 (ATM kinase inhibitor; *p* = 0.04), and Pevonedistat (NEDD8-activating enzyme (NAE) inhibitor; *p* = 0.03). On the other hand, *KMT2A*-r cell lines were more resistant to the γ-Secretase inhibitor, Avagacestat (*p* = 0.005) (Figure 4A). Due to availability of data from analyses of *ex-vivo* drug sensitivity to a variety of some small-molecule inhibitors, including Foretinib (Tyner et al., 2018), we also evaluated the IC50 values for this drug in AML samples of the Beat AML cohort (nine of 13 *KMT2A*-r and 247 of 390 *KMT2A*-WT). We did not observe significant differences between *KMT2A*-r and other molecular subgroups (Figure 4B). Interestingly, Foretinib is an ATP-competitive inhibitor of tyrosine kinases, and both acute leukemia cell lines with the lowest IC50 values to this drug carried *FLT3* activating mutations (MONO-MAC-6, p.V592A; MOLM-13, ITD). When we evaluated the IC50 values in the Beat AML data, we observed that samples with *FLT3* activating mutations were significantly more sensitive compared with *FLT3*-WT samples (Figure 4C).

In order to identify other potential drug-gene interactions, we uploaded a list with these eight drugs, whose *KMT2A*-r cell lines demonstrated sensitivity, in the DGIdb database. As result, 127 potential targets were found for three drugs (Fluorouracil, Foretinib, Pevonedistat) (Supplementary Table S9). Next, we evaluated if these targets were upregulated in *KMT2A*-r acute leukemia samples. We found *ALDH3A1*, *PIK3R2*, and *TYMP* expression levels increased in *KMT2A*-r samples when compared with *KMT2A*-WT (Figure 4D).

## Discussion

The identification of *KMT2A*-r at the molecular level is challenging, considering the high number of gene fusions attributed to this group of alterations (Meyer et al., 2018; Meyer et al., 2019). Back in 1996, Bernstein’s group produced a monoclonal antibody (named 7.1) that recognises the CSPG4/NG2, which is a chondroitin sulfate proteoglycan molecule (Smith et al., 1996). The CSPG4 antigen expression was associated with AML-M5 and 11q23 rearrangements, where *KMT2A* is located. Immunophenotyping of leukemia cells using the 7.1 antibody provided the possibility to estimate the occurrence of *KMT2A*-r. Further studies have confirmed the association between CSPG4 expression and *KMT2A*-r in acute leukemia (Wuchter et al., 2000; Schwartz et al., 2003). However, controversies were raised regarding the accuracy of prediction among different centers, age groups, and leukemia subtypes (Emerenciano et al., 2011; Menendez and Bueno, 2011). Here, we have performed machine-learning analyses to provide novel accurate markers for prediction of *KMT2A*-r in several types of acute leukemia, including AML, B-ALL, T-ALL, and ALAL.

Despite the variable frequency of *KMT2A*-r between each group of acute leukemia, the high number of gene fusions and dismal outcome of these patients instigated us to develop novel tools for the diagnosis of this disease (Winters and Bernt, 2017; Meyer et al., 2018). The unique gene expression profile observed in *KMT2A*-r points out one way for discriminating this genetic subtype from other alterations in ALL and AML (Armstrong et al., 2002). Thus, although CSPG4 expression has been widely used for the prediction of *KMT2A*-r so far, machine learning analyses using transcriptomic data currently allow us to agnostically identify novel markers associated with most acute leukemia cases with *KMT2A*-r. To our knowledge, this is the first study to investigate the application of machine learning for the prediction of *KMT2A*-r in acute leukemia. To construct our predictive algorithm, we used RNA-seq data as input, a single approach that can identify many alterations (e.g., fusion transcripts) and provide gene expression profiles. Although it is important to implement this technology for additional clinical benefits (Brown et al., 2020), it is still not used in clinical diagnostics for acute leukemia in many countries, and more accessible methods should be provided to benefit every patient with acute leukemia.

Here, we revealed 20 gene transcripts that, in combination, were able to predict *KMT2A*-r in acute leukemia. The two most important genes were *SKIDA1* and *LAMP5*. The *SKIDA1* gene (also named *DLN1*/*C10orf140*) is located at 10p12.31, in very close proximity to *MLLT10* (downstream) and ∼340 kb upstream *NEBL*. Both genes have already been found as partners of *KMT2A* fusions (Emerenciano et al., 2013; Meyer et al., 2018). Considering that previous studies have reported an increased expression of genes in the vicinity of *MLLT10* associated with *KMT2A*-*MLLT10*, but not other fusion partners in T-ALL (Dik et al., 2005; Kang et al., 2018), we also analyzed its expression among each *KMT2A* fusion included in this study. However, we observed that *SKIDA1* overexpression was not restricted to *KMT2A-MLLT10* positive patients. Because most of our patients were diagnosed with AML (13 out of 14 cases), we speculate that 10p12.2 rearrangements may disturb the expression of other genes within this region or this association could be restricted to T-ALL. Additionally, a recent murine study revealed that Skida1 might cooperate to sustain hematopoietic stem cells and hematopoietic committed progenitor cells presenting *KMT2A* fusion (Mendoza-Castrejon et al., 2021). The *LAMP5* gene is a member of the lysosomal associated membrane protein family, and it is located at the cytogenetic band 20p12.2. *LAMP5* is a direct target of the KMT2A fusion protein, which might activate it transcriptionally (Gracia-Maldonado et al., 2021). As a consequence, several studies identified *LAMP5* overexpression in *KMT2A*-r leukemia (Ross et al., 2004; Valk et al., 2004; Zangrando et al., 2009; Stam et al., 2010; Wang et al., 2019). While SKIDA1 protein localizes within the nucleus and cytosol, LAMP5 is associated with endosomes, lysosomes, and the plasma membrane.

The *SKIDA1* was the best predictor of *KMT2A*-r across all subtypes of acute leukemia, especially in B-ALL and T-ALL. Although extensive efforts have been made to evaluate the value of CSPG4 for the prediction of *KMT2A*-r, most studies were restricted to B-ALL and AML. Moreover, the sensitivity and specificity associated with CSPG4 expression in *KMT2A*-r acute leukemias is controversial, and many patients harboring *KMT2A*-r lack its expression (Menendez and Bueno, 2011). Additionally, previous studies have shown that *CSPG4* expression is correlated with the degree of maturation arrest in AML (Mauvieux et al., 1999), and higher among patients with AML-M5 (Petrovici et al., 2010). In this context, CSPG4 expression evidences *KMT2A*-r in the monoblastic population of AML, but fails to indicate this alteration especially for AML-M1 and -M2 (Mauvieux et al., 1999). As discussed, the leukemia phenotype may play a role in dictating CSPG4 expression, and the vast number of fusion transcripts associated with *KMT2A*-r could impact its expression levels. While *SKIDA1* was broadly overexpressed regardless of *KMT2A* fusion, high levels of *CSPG4* were quite restricted to *KMT2A-AFF1*, *KMT2A-MLLT10*, *KMT2A-MLLT3*, and *KMT2A-MLLT6*. Although Wuchter and collaborators have found no clear correlation between CSPG4 levels by immunophenotyping and *KMT2A* partners (Wuchter et al., 2000), diverse studies indicate its use in specific types of *KMT2A* fusions. For instance, the 7.1 antibody distinguished patients with t(4;11) or t(11;19) in childhood ALL (Behm et al., 1996), and another study revealed a lack of expression among t(10;11) and t(11;17) in AML (Hilden et al., 1997). Despite discordances regarding the relationship between CSPG4 expression and *KMT2A* fusions, differences in 7.1 reactivity in *KMT2A*-r ALL and AML supports the importance of the identification of alternative markers for prediction of *KMT2A*-r. We highlight that *SKIDA1* is one of the promising markers, which has consistent overexpression among several types of acute leukemia, and no evidence of biased expression toward one *KMT2A* fusion partner.

Screening methods for the identification of *KMT2A*-r are expected to be sensitive to detect all or most cases with this genetic alteration. This fact is even more important for rare *KMT2A* fusions, which are generally not evaluated at routine diagnostics by RT-PCR. Although FISH is one appropriate approach for detecting *KMT2A*-r in this population, recently we have demonstrated that several patients with *KMT2A* fusions within its minor breakpoint cluster region appear to be normal at FISH inspection using break apart probes (Meyer et al., 2019). For instance, *KMT2A-USP2* fusions are derived from an inversion within 11q23, and FISH is able to indicate *KMT2A*-r only for those cases accompanied by 3′*KMT2A* deletion. In this work, we observed that *CSPG4* expression was unable to predict all *KMT2A-USP2* fusions in B-ALL. Conversely, *LAMP5* expression was capable of estimating all of these fusions. Considering the accuracy of those markers for estimating common and rare *KMT2A*-r within our cohorts, we consider *SKIDA1* and *LAMP5* expression good predictors of *KMT2A*-r. *SKIDA1* expression may predict those rearrangements in most subtypes of acute leukemia, while *LAMP5* presents good performance to point out *KMT2A*-r in B-ALL and ALAL, including the *KMT2A-USP2* fusion. A recent study showed specific expression of LAMP5 on the cell surface of several *KMT2A*-r cell lines using flow cytometry, and demonstrated that its inhibition reduced cell viability (Gracia-Maldonado et al., 2021). Thus, LAMP5 may serve as both a marker and treatment target in *KMT2A*-r leukemia.

Over the last decades, a great effort has also been made by the scientific community in the identification of novel effective therapeutic approaches for *KMT2A*-r acute leukemias due to its association with poor response to standard chemotherapy. With this in mind, we combined IC50 data from several acute leukemia cell lines, drug-gene interaction information, besides transcriptomic analysis to search for new possibilities of targets for the treatment of *KMT2A*-r leukemia. Our results pointed, at first, to the foretinib (GSK1363089) as a new potential therapeutic agent for this leukemia subtype. Foretinib is an oral multikinase inhibitor targeting MET, RON, AXL, VEGFR, c-KIT, FLT3, and PDGFR signaling pathways. Based on that, we verified that patient’s cells harboring *FLT3* mutations, a frequent secondary event in *KMT2A*-r leukemia, showed higher sensitivity to Foretinib, suggesting that this drug might turn into a therapeutic option for these subgroups. Foretinib has already been used in several cancer treatment trials, including Hepatocellular Carcinoma (NCT00920192), Breast Cancer (NCT01147484, NCT01138384), Non-Small-Cell Lung Cancer (NCT02034097, NCT01068587), Solid Tumours (NCT00742131, NCT00742261, NCT00743067), Head and Neck (NCT00725764), Papillary Renal Cell Carcinoma (NCT00726323), and Gastric Carcinoma (NCT00725712). In leukemia, this drug induces mitotic catastrophe in chronic myelogenous leukemia (CML) cells *via* JNK-dependent inhibition of Plk1 expression and triggers apoptosis by a caspase 2-mediated mechanism (Dufies et al., 2011). In addition, Schneider and collaborators observed that *RAS*-mutant *KMT2A*-r ALL cells treated with DNA methyltransferase inhibitor decitabine in combination with the MEK inhibitor pimasertib strongly decreased cell viability compared to either drug alone. Besides, the combination of foretinib and decitabine also decreased cell viability and indicated moderate synergy in cell lines (Schneider et al., 2020). Although foretinib was not evaluated, Kampen and collaborators had already shown that together both MEK and VEGFR-2 inhibition can induce cell death in a subset of *KMT2A*-r AML primary samples (Kampen et al., 2014). We also demonstrated that *KMT2A*-r acute leukemia patients overexpress three genes (*ALDH3A1*, *PIK3R2*, and *TYMP*) with interaction annotation to 5-Fluorouracil. The upregulation of thymidine phosphorylase (*TYMP*) is commonly associated with 5-fluorouracil resistance (Watson et al., 2010; Peri et al., 2021). However, the TPI, a TYMP inhibitor approved by the US FDA, was associated with a better overall survival of refractory colorectal cancer patients (Mayer et al., 2015). It is important to highlight that *ALDH3A1* was also listed among the 247 genes selected for the machine learning model and has already been shown that its selective inhibition by other compounds could increase chemosensitivity in highly *ALDH3A1*-expressing tumors (Parajuli et al., 2014; Okazaki et al., 2018). *PIK3R2* is responsible for coding the subunit beta regulatory component of PI3K and could also be a potential target for the AZD6482 drug, a PI3Kβ inhibitor (Xu et al., 2019; Yang et al., 2019).

In this study, we observed that our algorithm delivered highly accurate predictions for *KMT2A*-r acute leukemias. Therefore, we were able to point out biomarkers (e.g., *SKIDA1* and *LAMP5*) to predict *KMT2A*-r regardless of acute leukemia subtypes. Further studies are needed in order to validate these markers and to translate their application into the routine diagnostics of acute leukemia, such as comparing its predictive value compared to CSPG4/NG2 by immunophenotyping. As a highlight, *LAMP5* expression was able to predict even the rare gene fusion *KMT2A-USP2*, which is often missed by routine methods, and that gene product also appears to be a potential treatment target in *KMT2A*-r leukemia. At last, our analysis suggested Foretinib as one therapeutic option for patients with *KMT2A*-r, especially those with AML and carrying *FLT3* activating mutations. Further clinical studies should now explore the application of this drug for the treatment of *KMT2A*-r acute leukemia.

## Data Availability

Publicly available datasets were analyzed in this study. This data can be found here: https://github.com/bioinformatics-inca/KMT2Ar-prediction (codes for machine learning analysis); https://portal.gdc.cancer.gov/projects (TARGET and TCGA datasets: accession TARGET-AML, TARGET-ALL-P2, TARGET-ALL-P3, and TCGA-LAML).
