# Supplementary material for: Novel Diagnostic and Therapeutic Options for KMT2A-Rearranged Acute Leukemias
Source: Front Pharmacol. 2022 Jun 6;13:749472. doi: 10.3389/fphar.2022.749472 (PMC9208280; doi:10.3389/fphar.2022.749472)
Supplement: Supplementary file 1 [file DataSheet1.docx]

***Supplementary Material***

# Supplementary Figures (Figures S1-S6)

#
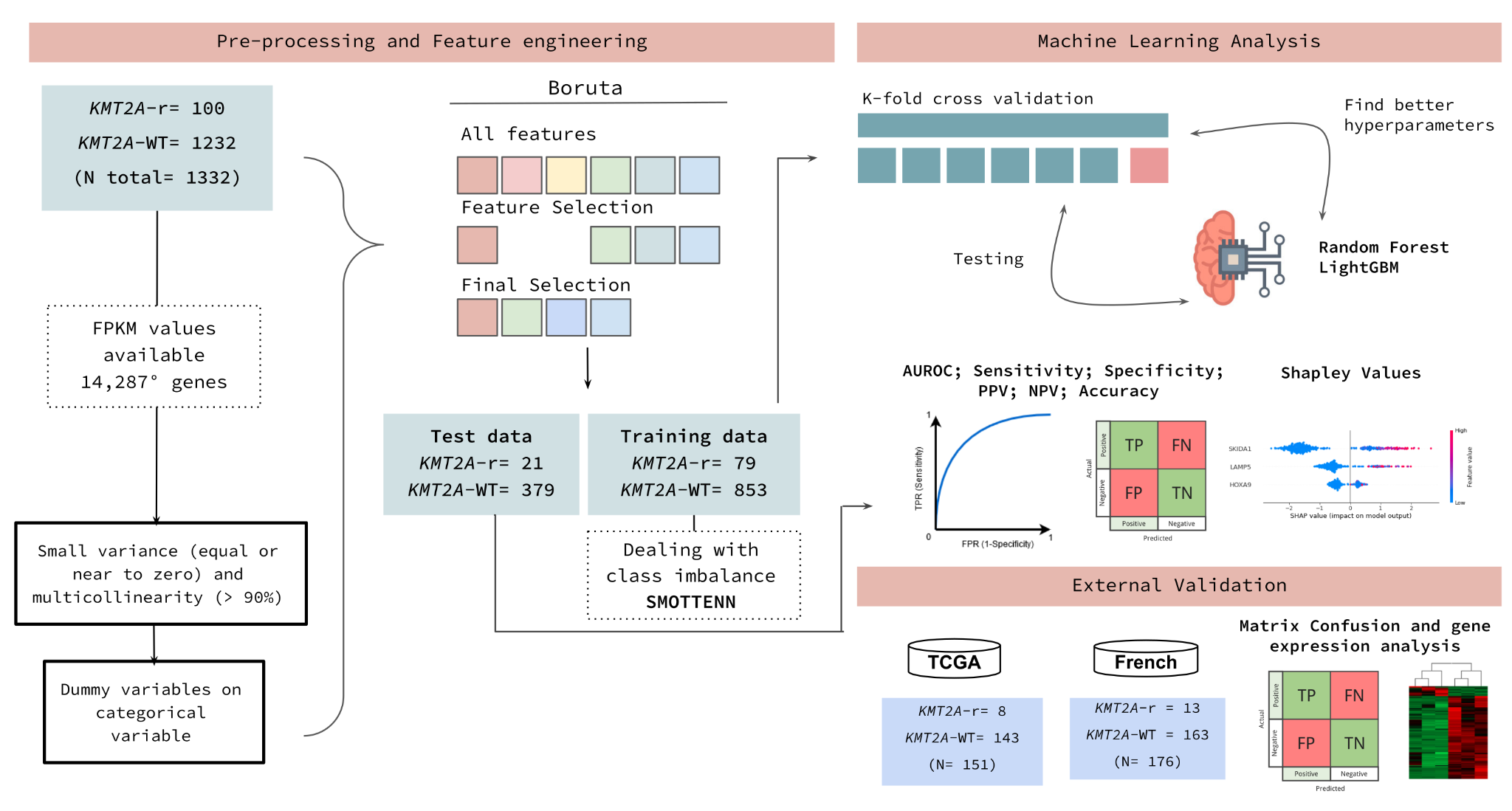
Figure S1. Workflow of machine learning analysis.

**
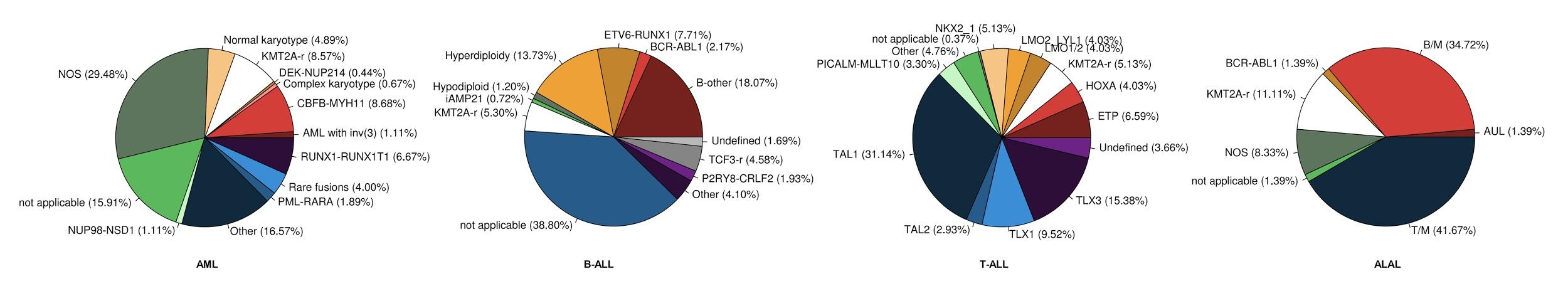
**

**Figure S2. Cytogenetic and molecular characterization of acute leukemia included in this work.** The pie charts illustrate the frequencies of the different cytogenetic/molecular subgroups in our entire cohort according to acute leukemia subtypes: AML, B-ALL, T-ALL, and ALAL. The *KMT2A*-r subgroup is highlighted in white boxes.

**
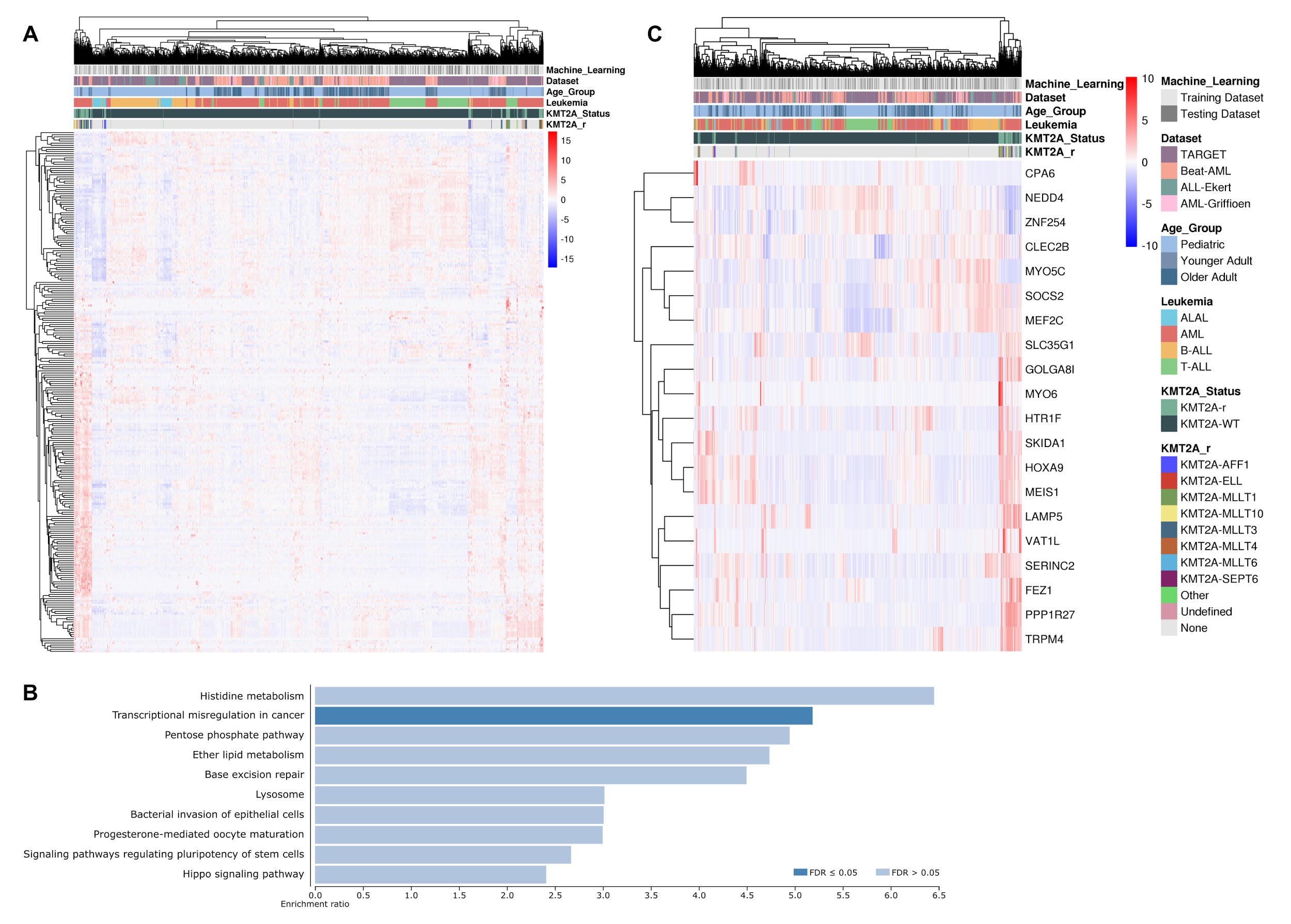
**

**Figure S3. Gene expression of *KMT2A*-r predictors.** (A) Heatmap showing the expression of 247 genes selected by our machine learning model, and (B) its pathway enrichment analysis. Dark blue bars represent significant pathways (FDR ≤ 0.05). (C) Heatmap illustrating the expression of top 20 genes with the greatest contribution for the prediction of *KMT2A*-r in the LightGBM algorithm model.


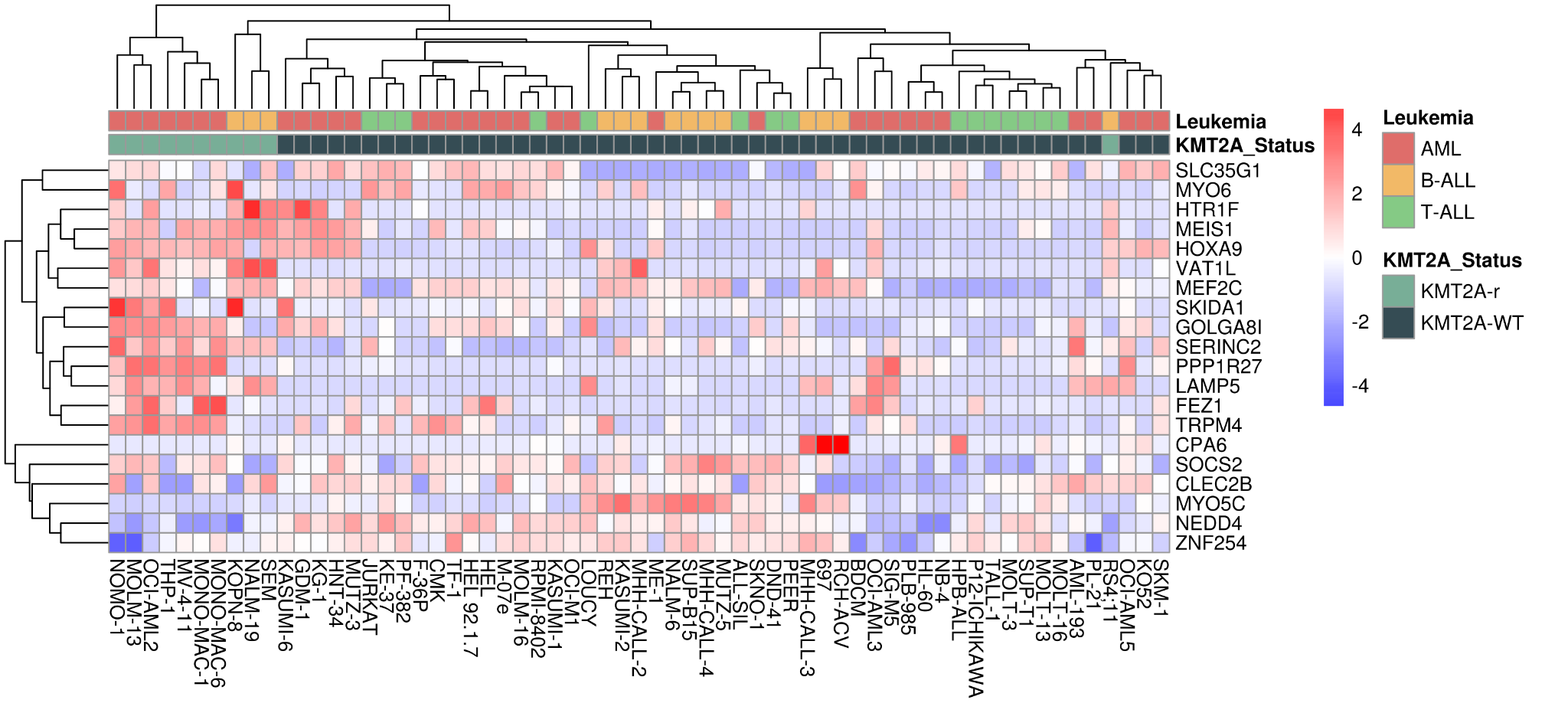


**Figure S4. Validation of *KMT2A*-r predictors in acute leukemia cell lines.** Heatmap showing the expression of top 20 genes selected by our final machine learning model in acute leukemia cell lines samples.


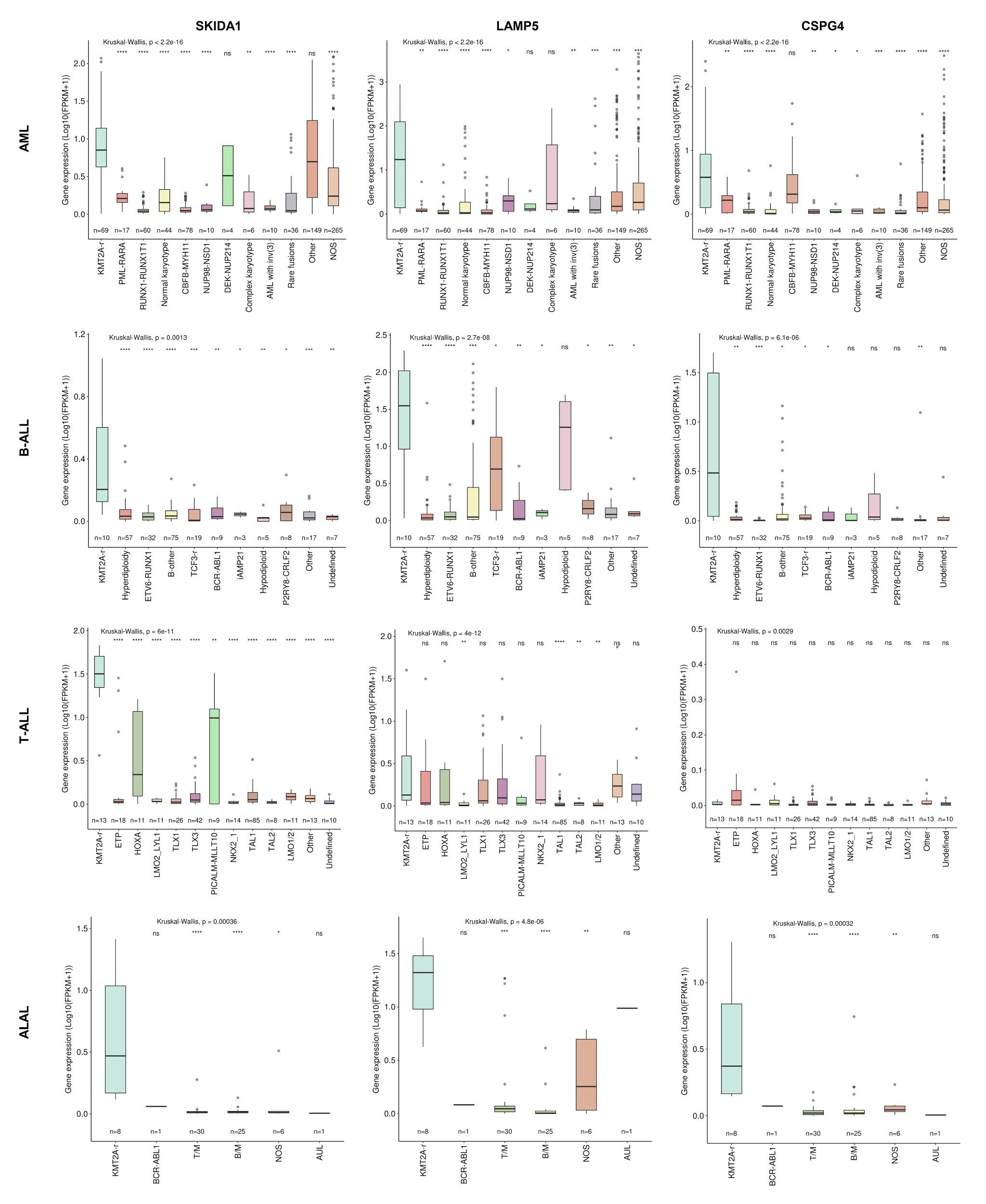


**Figure S5. Relationship between *SKIDA1*, *LAMP5*, and *CSPG4* expression and acute leukemia subgroups.** Transcript expression among cytogenetic/molecular subgroups in their respective acute leukemia subtypes. *KMT2A*-r was used as the reference group for statistical analyses. ns: *P* > 0.05; *: *P* ≤ 0.05; **: *P* ≤ 0.01; ***: *P* ≤ 0.001; ****: *P* ≤ 0.0001.


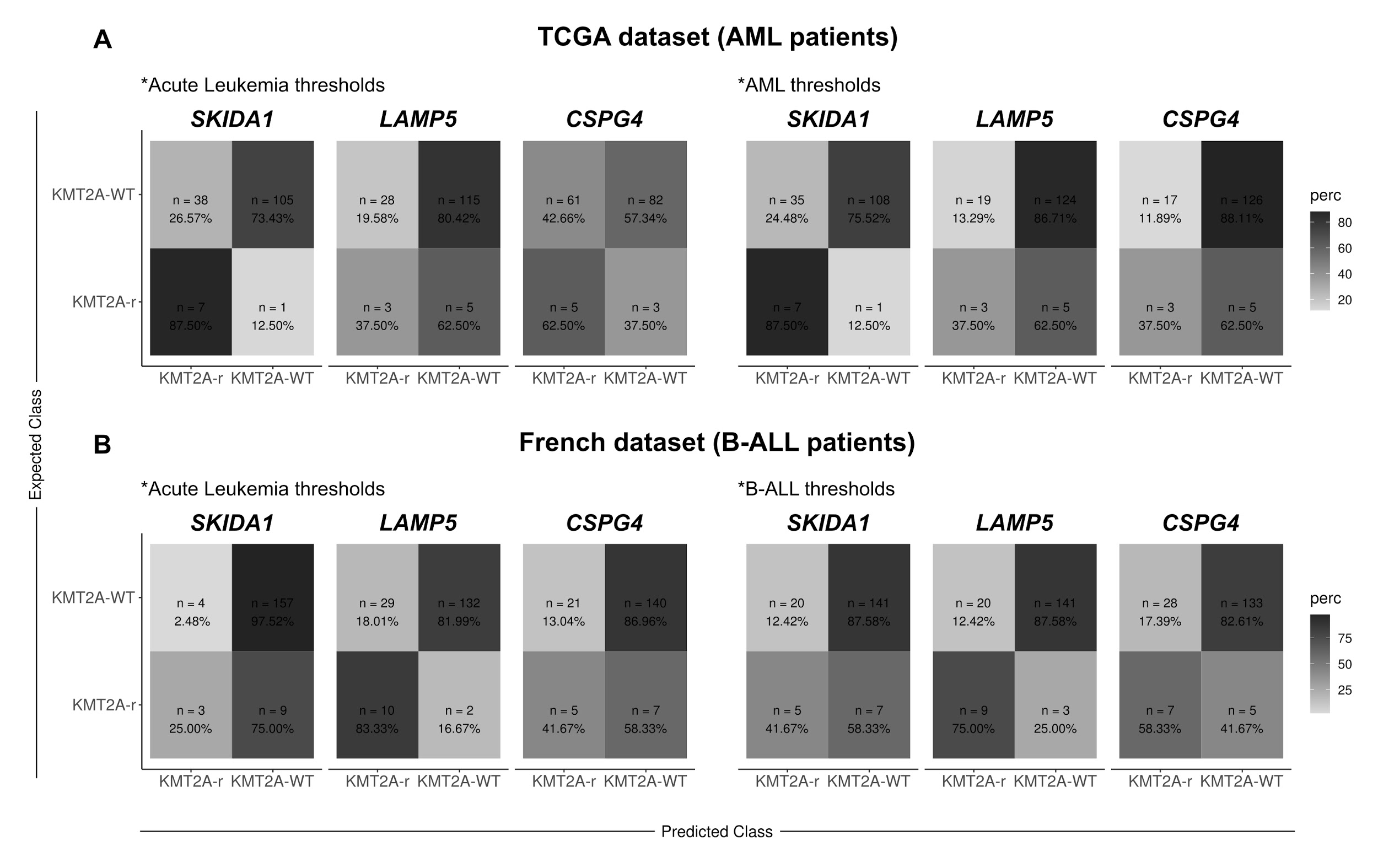


**Figure S6. Validation of potential biomarkers for *KMT2A*-r.** Confusion matrices demonstrating the predictive power of *SKIDA1*, *LAMP5* and *CSPG4* for *KMT2A*-r in the validation cohorts of (A) AML (TCGA dataset) and (B) ALL (French dataset) using their respective thresholds.

1. **Supplementary Tables (Tables S1-S9)**

Table S1. Description of datasets.

| **Cohorts** | | | | **Data (number of samples)** | | | ***KMT2A* status (number of samples)** | | **RNA-Seq** | |
| --- | --- | --- | --- | --- | --- | --- | --- | --- | --- | --- |
| **Datasets** | **Acute Leukemia Subtypes** | **Age group¹** | **Data Availability** | **With *KMT2A* status** | **Gene expression (RNA-Seq)** | **Both**  **(N final)** | ***KMT2A*-WT** | ***KMT2A*-r** | **Library construction** | **Platform** |
| **Train/test datasets** | | | |  |  | **N total = 1332** | **N total = 1232** | **N total = 100** |  |  |
| TARGET² | B-ALL | Pediatric | dbGaP accession phs000464 | 143 | 187 | 143 | 139 | 4 | Paired-end | Illumina HiSeq2000 |
|  | T-ALL |  | dbGaP accession phs000464 | 244 | 264 | 244 | 232 | 12 | Paired-end | Illumina HiSeq2000 |
|  | AML |  | dbGaP accession phs000465 | 957 | 256 | 248 | 199 | 49 | Paired-end | Illumina HiSeq2500 |
|  | ALAL |  | dbGaP accession phs000464 | 71 | 87 | 71 | 63 | 8 | Paired-end | Illumina HiSeq2500 |
| Beat AML | AML | Pediatric and adult | dbGaP accession phs001657.v1.p1 | 609 | 451 | 403 | 390 | 13 | Paired-end | Illumina HiSeq2500 |
| ALL-Ekert³ | B-ALL | Pediatric | EGA accession EGAS00001004212 | 99 | 99 | 99 | 93 | 6 | Paired-end | Illumina HiSeq4000 |
|  | T-ALL |  |  | 27 | 27 | 27 | 26 | 1 |  |  |
| AML-Griffioen³ | AML | Adult | EGA accession EGAS00001003096 | 100 | 98 | 97 | 90 | 7 | Paired-end | Illumina HiSeq2500 |
| **External validation datasets** | | | |  |  | **N total = 327** | **N total = 306** | **N total = 21** |  |  |
| TCGA | AML | Pediatric and adult | dbGaP accession phs000178 | 200 | 151 | 151 | 143 | 8 | Paired-end | Illumina HiSeq2000 |
| French | B-ALL | Pediatric | Upon request | 173 | 173 | 173 | 161 | 12 | Paired-end | Illumina NovaSeq |
|  | T-ALL |  |  | 2 | 2 | 2 | 1 | 1 |  |  |
|  | ALAL |  |  | 1 | 1 | 1 | 1 | 0 |  |  |
| **TOTAL** | **-** |  | **-** | **-** | **-** | **1659** | **1538** | **121** | **-** | **-** |

¹ Pediatric (<22y); Adult - younger adults (≥22y and <60y) and older adults (≥60y).

² Data was retrieved from TARGET ALL Phase II discovery cohort (B-ALL), TARGET ALL Phase II validation cohort (T-ALL), TARGET AML discovery cohort (AML) and TARGET ALL Phase III subproject (ALAL).

³ Fictitious dataset name: leukemia subtype-corresponding author's surname.

Table S2. Cohort characterization according to training and testing datasets.

| **Variables** |  | **Training Dataset** | | | | | |  | **Testing Dataset** | | | | | |  | ***P*-value*** |
| --- | --- | --- | --- | --- | --- | --- | --- | --- | --- | --- | --- | --- | --- | --- | --- | --- |
|  |  | **Overall** | | ***KMT2A*-r** | | ***KMT2A*-WT** | |  | **Overall** | | ***KMT2A*-r** | | ***KMT2A*-WT** | |  |  |
| *Age group¹* |  |  |  |  |  |  |  |  |  |  |  |  |  |  |  | 0.643 |
| Pediatric |  | 596 | (63.9) | 63 | (79.7) | 533 | (62.5) |  | 247 | (61.8) | 16 | (76.2) | 231 | (60.9) |  |  |
| Younger adult |  | 167 | (17.9) | 15 | (19.0) | 152 | (17.8) |  | 72 | (18.0) | 4 | (19.0) | 68 | (17.9) |  |  |
| Older adult |  | 169 | (18.1) | 1 | (1.3) | 168 | (19.7) |  | 81 | (20.3) | 1 | (4.8) | 80 | (21.1) |  |  |
| *Sex* |  |  |  |  |  |  |  |  |  |  |  |  |  |  |  | 0.900 |
| Female |  | 384 | (41.2) | 42 | (53.2) | 342 | (40.1) |  | 167 | (41.8) | 10 | (47.6) | 157 | (41.4) |  |  |
| Male |  | 548 | (58.8) | 37 | (46.8) | 511 | (59.9) |  | 233 | (58.3) | 11 | (52.4) | 222 | (58.6) |  |  |
| *Sample type* |  |  |  |  |  |  |  |  |  |  |  |  |  |  |  | 0.532 |
| Diagnosis |  | 836 | (89.7) | 77 | (97.5) | 759 | (89.0) |  | 357 | (89.3) | 21 | (100.0) | 336 | (88.7) |  |  |
| Refractory |  | 65 | (7.0) | 1 | (1.3) | 64 | (7.5) |  | 28 | (7.0) | 0 | (0.0) | 28 | (7.4) |  |  |
| Relapse |  | 14 | (1.5) | 1 | (1.3) | 13 | (1.5) |  | 9 | (2.3) | 0 | (0.0) | 9 | (2.4) |  |  |
| Remission |  | 12 | (1.3) | 0 | (0.0) | 12 | (1.4) |  | 6 | (1.5) | 0 | (0.0) | 6 | (1.6) |  |  |
| Unknown |  | 5 | (0.5) | 0 | (0.0) | 5 | (0.6) |  | 0 | (0.0) | 0 | (0.0) | 0 | (0.0) |  |  |
| *Leukemia Subtype* |  |  |  |  |  |  |  |  |  |  |  |  |  |  |  | 0.163 |
| B-ALL |  | 157 | (16.8) | 8 | (10.1) | 149 | (17.5) |  | 85 | (21.3) | 2 | (9.5) | 83 | (21.9) |  |  |
| T-ALL |  | 192 | (20.6) | 11 | (13.9) | 181 | (21.2) |  | 79 | (19.8) | 2 | (9.5) | 77 | (20.3) |  |  |
| AML |  | 528 | (56.7) | 53 | (67.1) | 475 | (55.7) |  | 220 | (55.0) | 16 | (76.2) | 204 | (53.8) |  |  |
| ALAL |  | 55 | (5.9) | 7 | (8.9) | 48 | (5.6) |  | 16 | (4.0) | 1 | (4.8) | 15 | (4.0) |  |  |
| *KMT2A status* |  |  |  |  |  |  |  |  |  |  |  |  |  |  |  | 0.053 |
| *KMT2A*-r |  | 79 | (8.5) | - | | - | |  | 21 | (5.3) | - | | - | |  |  |
| *KMT2A*-WT |  | 853 | (91.5) | - | | - | |  | 379 | (94.8) | - | | - | |  |  |
| **TOTAL** |  | **932** | **(100.0)** | **79** | **(100.0)** | **853** | **(100.0)** |  | **400** | **(100.0)** | **21** | **(100.0)** | **379** | **(100.0)** |  | **-** |

* Pearson's Chi-squared test.

¹ Pediatric (<22y); Adult - younger adults (≥22y and <60y) and older adults (≥60y).

Table S3. Cytogenetic/molecular alterations according to training and testing datasets.

| **Subgroups** |  | **Training Dataset** | |  | **Testing Dataset** | |
| --- | --- | --- | --- | --- | --- | --- |
| *AML* |  |  |  |  |  |  |
| AML with inv(3) |  | 6 | (1.1) |  | 4 | (1.8) |
| *PML-RARA* |  | 14 | (2.7) |  | 3 | (1.4) |
| *CBFB-MYH11* |  | 61 | (11.6) |  | 17 | (7.7) |
| *RUNX1-RUNX1T1* |  | 43 | (8.1) |  | 17 | (7.7) |
| *KMT2A*-r |  | 53 | (10.0) |  | 16 | (7.3) |
| Normal karyotype |  | 30 | (5.7) |  | 14 | (6.4) |
| Rare fusions |  | 23 | (4.4) |  | 13 | (5.9) |
| *DEK-NUP214* |  | 3 | (0.6) |  | 1 | (0.5) |
| *NUP98-NSD1* |  | 8 | (1.5) |  | 2 | (0.9) |
| Complex karyotype |  | 5 | (0.9) |  | 1 | (0.5) |
| Other |  | 100 | (18.9) |  | 49 | (22.3) |
| NOS |  | 182 | (34.5) |  | 83 | (37.7) |
| *B-ALL* |  |  |  |  |  |  |
| B-other |  | 49 | (31.2) |  | 26 | (30.6) |
| Hyperdiploidy |  | 37 | (23.6) |  | 20 | (23.5) |
| *TCF3-r* |  | 13 | (8.3) |  | 6 | (7.1) |
| *ETV6-RUNX1* |  | 19 | (12.1) |  | 13 | (15.3) |
| *KMT2A*-r |  | 8 | (5.1) |  | 2 | (2.4) |
| *BCR-ABL1* |  | 3 | (1.9) |  | 6 | (7.1) |
| iAMP21 |  | 2 | (1.3) |  | 1 | (1.2) |
| *P2RY8-CRLF2* |  | 7 | (4.5) |  | 1 | (1.2) |
| Hypodiploidy |  | 3 | (1.9) |  | 2 | (2.4) |
| Other |  | 11 | (7.0) |  | 6 | (7.1) |
| Undefined |  | 5 | (3.2) |  | 2 | (2.4) |
| *T-ALL* |  |  |  |  |  |  |
| *TAL1* |  | 56 | (29.2) |  | 29 | (36.7) |
| *TLX3* |  | 33 | (17.2) |  | 9 | (11.4) |
| *TLX1* |  | 20 | (10.4) |  | 6 | (7.6) |
| ETP |  | 10 | (5.2) |  | 8 | (10.1) |
| *NKX2_1* |  | 10 | (5.2) |  | 4 | (5.1) |
| *LMO2_LYL1* |  | 9 | (4.7) |  | 2 | (2.5) |
| *KMT2A*-r |  | 11 | (5.7) |  | 2 | (2.5) |
| *LMO1/2* |  | 7 | (3.6) |  | 4 | (5.1) |
| *TAL2* |  | 4 | (2.1) |  | 4 | (5.1) |
| *PICALM-MLLT10* |  | 5 | (2.6) |  | 4 | (5.1) |
| *HOXA* |  | 8 | (4.2) |  | 3 | (3.8) |
| Other |  | 9 | (4.7) |  | 4 | (5.1) |
| Undefined |  | 10 | (5.2) |  | 0 | (0.0) |
| *ALAL* |  |  |  |  |  |  |
| T/M |  | 25 | (45.5) |  | 5 | (31.3) |
| B/M |  | 17 | (30.9) |  | 8 | (50.0) |
| *KMT2A*-r |  | 7 | (12.7) |  | 1 | (6.3) |
| NOS |  | 5 | (9.1) |  | 1 | (6.3) |
| *BCR-ABL1* |  | 0 | (0.0) |  | 1 | (6.3) |
| AUL |  | 1 | (1.8) |  | 0 | (0.0) |
| **TOTAL** |  | **932** | **(100.0)** |  | **400** | **(100.0)** |

Table S4a. Performance measures of machine learning models.

| Performance measures* |  | **Random Forest** | | |  |  | **LightGBM** | | | |
| --- | --- | --- | --- | --- | --- | --- | --- | --- | --- | --- |
|  |  | 247 genes without clinical variables | 247 genes with clinical variables | 247 genes with Age group variable | 247 genes with Leukemia variable |  | **247 genes without clinical variables** | 247 genes with clinical variables | 247 genes with Age group variable | 247 genes with Leukemia variable |
| AUC |  | 0.984 | 0.985 | 0.987 | 0.984 |  | **0.988** | 0.989 | 0.988 | 0.988 |
| Accuracy |  | 0.970 | 0.975 | 0.973 | 0.970 |  | **0.973** | 0.970 | 0.970 | 0.970 |
| Sensitivity |  | 0.810 | 0.857 | 0.810 | 0.810 |  | **0.905** | 0.905 | 0.905 | 0.857 |
| Specificity |  | 0.979 | 0.982 | 0.982 | 0.979 |  | **0.976** | 0.974 | 0.974 | 0.976 |
| PPV |  | 0.680 | 0.720 | 0.708 | 0.680 |  | **0.679** | 0.655 | 0.655 | 0.667 |
| NPV |  | 0.989 | 0.992 | 0.989 | 0.989 |  | **0.995** | 0.995 | 0.995 | 0.992 |

* *KMT2A*-r as “positive” class

Table S4b. Performance measures of machine learning models on Top 20 genes.

| Performance measures* |  | **LightGBM** | | |  |
| --- | --- | --- | --- | --- | --- |
|  |  | 20 genes without clinical variables | 20 genes with clinical variables | 20 genes with Age group variable | **20 genes with Leukemia variable**** |
| AUC |  | 0.979 | 0.985 | 0.978 | **0.984** |
| Accuracy |  | 0.965 | 0.970 | 0.973 | **0.970** |
| Sensitivity |  | 0.905 | 0.905 | 0.905 | **0.905** |
| Specificity |  | 0.968 | 0.974 | 0.976 | **0.974** |
| PPV |  | 0.613 | 0.655 | 0.679 | **0.655** |
| NPV |  | 0.995 | 0.995 | 0.995 | **0.995** |

* *KMT2A*-r as “positive” class

** Performed better when validated using data obtained from the TCGA (AUC: 0.868)"

Table S5. Information about the false negative/positive groups.

| **Patient ID** | **Type error** | ***KMT2A* Status** | ***KMT2A*-r** | **Leukemia Subtype** | **Molecular Subgroup** | **Machine Learning Dataset** | **Study** | **Age (in years)** | **Sex** |
| --- | --- | --- | --- | --- | --- | --- | --- | --- | --- |
| TARGET-20-PASWAT | False negative | *KMT2A*-r | *KMT2A-SEPT6* | AML | *KMT2A*-r | Test | TARGET | 14.1 | Female |
| TARGET-10-PASZJW | False negative | *KMT2A*-r | *KMT2A-CT45A4* | T-ALL | *KMT2A*-r | Test | TARGET | 30.0 | Male |
| TCGA-AB-2834 | False negative | *KMT2A*-r | *KMT2A-ELL* | AML | *KMT2A*-r | Validation | TCGA | 33.0 | Male |
| TCGA-AB-2844 | False negative | *KMT2A*-r | *KMT2A-ELL* | AML | *KMT2A*-r | Validation | TCGA | 63.0 | Male |
| 21597 | False negative* | *KMT2A*-r | *KMT2A-USP2* | B-ALL | *KMT2A*-r | Validation | French | 0.7 | Female |
| 26181 | False negative* | *KMT2A*-r | *KMT2A*-7q22.3 | B-ALL | *KMT2A*-r | Validation | French | 6.6 | Male |
| 29468 | False negative* | *KMT2A*-r | *KMT2A-MGMT* | B-ALL | *KMT2A*-r | Validation | French | 9.3 | Male |
| TARGET-20-PANGJY | False positive | *KMT2A*-WT | None | AML | Other | Test | TARGET | 1.6 | Female |
| TARGET-20-PASBPK | False positive | *KMT2A*-WT | None | AML | Other | Test | TARGET | 0.4 | Male |
| TARGET-10-PANYEJ | False positive | *KMT2A*-WT | None | B-ALL | Hyperdiploidy | Test | TARGET | 1.9 | Male |
| TARGET-10-PATKYI | False positive | *KMT2A*-WT | None | T-ALL | ETP | Test | TARGET | 20.9 | Male |
| 13-00255 | False positive | *KMT2A*-WT | None | AML | NOS | Test | Beat-AML | 72.0 | Male |
| 14-00012 | False positive | *KMT2A*-WT | None | AML | Other | Test | Beat-AML | 72.0 | Male |
| 14-00231 | False positive | *KMT2A*-WT | None | AML | NOS | Test | Beat-AML | 57.0 | Female |
| 15-00563 | False positive | *KMT2A*-WT | None | AML | NOS | Test | Beat-AML | 2.0 | Female |
| 16-00951 | False positive | *KMT2A*-WT | None | AML | NOS | Test | Beat-AML | 42.0 | Male |
| 3-032 | False positive | *KMT2A*-WT | None | AML | Other | Test | EGA-AML | 58.0 | Female |
| TCGA-AB-2925 | False positive | *KMT2A*-WT | None | AML | Not applicable | Validation | TCGA | 57.0 | Male |
| TCGA-AB-2861 | False positive | *KMT2A*-WT | None | AML | Not applicable | Validation | TCGA | 76.0 | Male |
| 27635 | False positive* | *KMT2A*-WT | None | B-ALL | Not applicable | Validation | French | 0.3 | Male |
| 23591 | False positive* | *KMT2A*-WT | None | B-ALL | Not applicable | Validation | French | 0.4 | Female |
| 27473 | False positive* | *KMT2A*-WT | None | B-ALL | Not applicable | Validation | French | 0.5 | Male |
| 27181 | False positive* | *KMT2A*-WT | None | B-ALL | Not applicable | Validation | French | 0.7 | Male |
| 27865 | False positive* | *KMT2A*-WT | None | B-ALL | Not applicable | Validation | French | 0.7 | Male |
| 24088 | False positive* | *KMT2A*-WT | None | B-ALL | Not applicable | Validation | French | 0.8 | Female |
| 27769 | False positive* | *KMT2A*-WT | None | B-ALL | Not applicable | Validation | French | 1.7 | Female |
| 28011 | False positive* | *KMT2A*-WT | None | B-ALL | Not applicable | Validation | French | 3.1 | Female |
| 26302 | False positive* | *KMT2A*-WT | None | B-ALL | Not applicable | Validation | French | 10.8 | Male |
| 27150 | False positive* | *KMT2A*-WT | None | B-ALL | Not applicable | Validation | French | 11.0 | Female |

*Type error considered based on hierarchical clustering (Figure 2D)

Table S6. Performance measures by single genes in TCGA and French datasets.

| Performance measures* |  | **AML patients (TCGA)** | | |  | **B-ALL patients (French)** | | |
| --- | --- | --- | --- | --- | --- | --- | --- | --- |
|  |  | *SKIDA1* | *LAMP5* | *CSPG4* |  | *SKIDA1* | *LAMP5* | *CSPG4* |
| The overall acute leukemia threshold | | | | | | | | |
| Accuracy |  | 0.742 | 0.781 | 0.576 |  | 0.925 | 0.821 | 0.838 |
| Sensitivity |  | 0.875 | 0.375 | 0.625 |  | 0.250 | 0.833 | 0.417 |
| Specificity |  | 0.734 | 0.802 | 0.573 |  | 0.975 | 0.820 | 0.870 |
| PPV |  | 0.155 | 0.097 | 0.076 |  | 0.429 | 0.256 | 0.192 |
| NPV |  | 0.990 | 0.958 | 0.965 |  | 0.946 | 0.985 | 0.952 |
| The respective acute leukemia subtype threshold (AML or B-ALL) | | | | | | | | |
| Accuracy |  | 0.762 | 0.841 | 0.854 |  | 0.844 | 0.867 | 0.810 |
| Sensitivity |  | 0.875 | 0.375 | 0.375 |  | 0.417 | 0.750 | 0.583 |
| Specificity |  | 0.755 | 0.867 | 0.881 |  | 0.876 | 0.876 | 0.826 |
| PPV |  | 0.167 | 0.136 | 0.150 |  | 0.200 | 0.310 | 0.200 |
| NPV |  | 0.991 | 0.961 | 0.962 |  | 0.953 | 0.980 | 0.964 |

* *KMT2A*-r as “positive” class

Table S7. *KMT2A* fusions according to acute leukemia subtypes in machine learning datasets.

| **Acute leukemia** | ***KMT2A* status** | ***KMT2A* fusions** |
| --- | --- | --- |
| AML | *KMT2A*-r (n = 69) | *KMT2A-MLLT3* (n = 35) |
|  | *KMT2A*-WT (n = 679) | *KMT2A-MLLT10* (n = 13) |
|  |  | *KMT2A-MLLT4* (n = 7) |
|  |  | *KMT2A-ELL* (n = 4) |
|  |  | *KMT2A-MLLT1* (n = 2) |
|  |  | *KMT2A-SEPT6* (n = 2) |
|  |  | *KMT2A-AFF1* (n = 1) |
|  |  | *KMT2A-CEP170B* (n = 1) |
|  |  | *KMT2A-MLLT6* (n = 1) |
|  |  | other *KMT2A* (n = 3) |
|  |  |  |
| B-ALL | *KMT2A*-r (n = 10) | *KMT2A-AFF1* (n = 3) |
|  | *KMT2A*-WT (n = 232) | *KMT2A-SARNP* (n = 1) |
|  |  | *KMT2A-USP2* (n = 1) |
|  |  | Undefined (n = 5) |
|  |  |  |
| T-ALL | *KMT2A*-r (n = 13) | *KMT2A-MLLT1* (n = 8) |
|  | *KMT2A*-WT (n = 258) | *KMT2A-CT45A4* (n = 1) |
|  |  | *KMT2A-ELL* (n = 1) |
|  |  | *KMT2A-MLLT4* (n = 1) |
|  |  | *KMT2A-MLLT6* (n = 1) |
|  |  | *KMT2A-MLLT10* (n = 1) |
|  |  |  |
| ALAL | *KMT2A*-r (n = 8) | *KMT2A-AFF1* (n = 5) |
|  | *KMT2A*-WT (n = 63) | *KMT2A-TET1* (n = 1) |
|  |  | other *KMT2A* (n = 2) |

Table S8. Acute leukemia cell lines included in this study from GDSC database.

| ***KMT2A* status** | **Acute leukemia** | **Cell line name (*KMT2A* fusion)** | |
| --- | --- | --- | --- |
| *KMT2A*-r (n = 6) | B-ALL (n = 1) | RS4;11 (*KMT2A-AFF1*) | |
|  | AML (n = 5) | MOLM-13 (*KMT2A-MLLT3*) | |
|  |  | MONO-MAC-6 (*KMT2A-MLLT3*) | |
|  |  | NOMO-1 (*KMT2A-MLLT3*) | |
|  |  | OCI-AML2 (*KMT2A-MLLT4*) | |
|  |  | THP-1 (*KMT2A-MLLT3*) | |
|  |  |  |  |
| *KMT2A*-WT (n = 27) | B-ALL (n = 1) | SUP-B15 |  |
|  | T-ALL (n = 11) | ALL-SIL | P12-ICHIKAWA |
|  |  | DND-41 | PF-382 |
|  |  | Jurkat | RPMI-8402 |
|  |  | KE-37 | SUP-T1 |
|  |  | MOLT-13 | TALL-1 |
|  |  | MOLT-16 |  |
|  | AML (n = 15) | CMK | NB4 |
|  |  | GDM-1 | OCI-AML3 |
|  |  | HEL | OCI-AML5 |
|  |  | HL-60 | OCI-M1 |
|  |  | KASUMI-1 | P31-FUJ |
|  |  | KG-1 | PL-21 |
|  |  | ME-1 | SIG-M5 |
|  |  | MOLM-16 |  |

Table S9. Drug-gene interactions according to DGIdb database.

| **Drug** | **Genes** | | | |
| --- | --- | --- | --- | --- |
| Fluorouracil | SELE | PERP | GSTP1 | CDC25C |
|  | VEGFA | EXO1 | CYP19A1 | ERBB2 |
|  | PTEN | DPYS | MUC2 | RGS5 |
|  | CYP2C19 | BIRC5 | CHN2 | TPT1 |
|  | ABCB1 | ENOSF1 | TP53AIP1 | RBX1 |
|  | PTPRC | DPYD | MEGF11 | ITGAL |
|  | KRAS | IRS1 | ERCC1 | IGFBP3 |
|  | XRCC1 | HLA-G | HSPA5 | CBR3 |
|  | ALDH3A1 | SHMT1 | FOXO1 | PON1 |
|  | FPGS | ABCG1 | CYP3A4 | DKK1 |
|  | KLC1 | REV3L | ABCG2 | FAS |
|  | EGFR | CYP2A6 | MET | PARD3B |
|  | UMPS | DTYMK | IL2RA | AURKA |
|  | CYP2C8 | TOP2A | CDKN1A | ATM |
|  | APC | NOS1 | PIK3CA | HMMR |
|  | HBB | TMEM167A | CYP1B1 | NT5C1A |
|  | CYP2E1 | TYMP | CBLB | LGR5 |
|  | TP53 | GNAS | BDNF | INSR |
|  | CCND1 | XRCC4 | MTHFR | NQO1 |
|  | PIK3R2 | ALCAM | MIR27A | CBR1 |
|  | XRCC3 | IL6R | FGFR4 | SMAD4 |
|  | DLG5 | SLCO1B1 | NOS3 | BRAF |
|  | WNT5B | ABCC5 | ABCC4 |  |
|  | IL11 | TSHB | CCNK |  |
| Foretinib | KDR | TEK | KIT | MST1R |
|  | FLT4 | FLT3 | FLT1 | YES1 |
|  | PDGFRA | MET | PDGFRB | ROS1 |
| Pevonedistat | UBA3 | NAE1 | SAE1 | UBA2 |

1. **Supplementary File (File S1)**

**File S1. Feature selection by the Boruta method**

**Keep:** ['PQLC2', 'COL9A2', 'AKR7A2', 'PTPRU', 'IL12RB2', 'BMP8B', 'STMN1', 'TXNDC12', 'C1orf54', 'FCGR1A', 'MCOLN2', 'FAM213B', 'CACHD1', 'SV2A', 'WDR63', 'SLC22A15', 'SERINC2', 'LPAR3', 'RNPEP', 'CLCNKA', 'RNF220', 'SPAG6', 'NEBL', 'MLLT10', 'DNTT', 'LZTS2', 'CISD1', 'DNAJC1', 'COMMD3', 'ZEB1', 'IPMK', 'REEP3', 'ZNF503', 'SLC35G1', 'FZD8', 'SKIDA1', 'KIAA1598', 'CASC10', 'DNAJC9', 'TMEM109', 'HIPK3', 'KIAA1549L', 'DPF2', 'ZC3H12C', 'FEZ1', 'TPP1', 'VPS37C', 'PRKCDBP', 'PLA2G16', 'RAB39A', 'CADM1', 'SVIP', 'SLC6A13', 'CD4', 'BCAT1', 'NUAK1', 'CLEC2B', 'LIN7A', 'ACRBP', 'SOCS2', 'BHLHE41', 'ITGA7', 'FGD4', 'CACNA2D4', 'PLBD2', 'CRADD', 'WNT10B', 'POLE', 'SMAD9', 'DACH1', 'PRKCH', 'SLC22A17', 'HIF1A', 'RIPK3', 'PARP2', 'OXA1L', 'NDRG2', 'CDCA4', 'NEDD4', 'CTSH', 'TGM5', 'MYO5C', 'GOLGA8I', 'LIPC', 'SPINT1', 'NIPA1', 'CSPG4', 'THSD4', 'KIF22', 'SYT17', 'ITGAX', 'NUDT7', 'ADCY9', 'LDHD', 'VAT1L', 'MT1F', 'CES1', 'GPR56', 'ALDH3A1', 'SCPEP1', 'C17orf53', 'KAT7', 'SLC47A1', 'CCL23', 'CBX2', 'PPP1R27', 'LRRC37B', 'ZNF532', 'MAPRE2', 'ZNF521', 'PAFAH1B3', 'TNNT1', 'PLD3', 'GRIN2D', 'ZNF85', 'ZFP30', 'ZSCAN18', 'C3', 'IGFLR1', 'TRPM4', 'ZNF331', 'KCNC3', 'ZNF776', 'ZNF256', 'BRSK1', 'FBXO27', 'ZNF91', 'HSD11B1L', 'ZNF571', 'ZNF329', 'LILRB4', 'ZNF681', 'ZNF418', 'ZNF682', 'ZNF772', 'ZNF461', 'ZNF544', 'ZNF43', 'ZNF254', 'ZNF134', 'ZNF844', 'ZNF737', 'ADAM23', 'TANC1', 'SPR', 'TGFBRAP1', 'DTNB', 'MEIS1', 'HNMT', 'AP1S3', 'B3GNT7', 'BRE', 'DAPL1', 'NMUR1', 'WDSUB1', 'MRPL33', 'PHACTR3', 'BFSP1', 'LAMP5', 'BTBD3', 'ZNF512B', 'SLC2A10', 'ZBTB21', 'UMODL1', 'CLTCL1', 'HMGXB4', 'UPK3A', 'CDC42EP1', 'IGF2BP2', 'PLCH1', 'ATG3', 'MBNL1', 'MRAS', 'CLSTN2', 'MAGEF1', 'HTR1F', 'VGLL3', 'PROM1', 'GUCY1B3', 'GLRB', 'TMEM156', 'CPEB2', 'MMRN1', 'OCIAD2', 'STK32B', 'GUCY1A3', 'RHOH', 'SMAD1', 'UNC5C', 'HEXB', 'MEF2C', 'NPR3', 'WWC1', 'MSX2', 'NRG2', 'RHOBTB3', 'IRX1', 'S100Z', 'ANXA2R', 'PRR16', 'BVES', 'WASF1', 'GPR126', 'QKI', 'SENP6', 'RUNX2', 'IL22RA2', 'NKAIN2', 'SUPT3H', 'MYO6', 'CD2AP', 'L3MBTL3', 'PPP1R3G', 'ETV1', 'HOXA9', 'ZC3HAV1', 'HOXA3', 'HOXA5', 'HOXA6', 'ABHD11', 'HOXA7', 'DYNC1I1', 'CLCN1', 'HOXA4', 'HOXA10', 'TRPS1', 'DNAJC5B', 'CPA6', 'FAM110B', 'PENK', 'CLDN23', 'VLDLR', 'GNA14', 'CDK20', 'PPAPDC3', 'PSIP1', 'FBP1', 'PBX3', 'GAS1', 'TDRD7', 'TKTL1', 'NXT2', 'FAM127A', 'CCNB3', 'RAB39B', 'KCNE1L', 'SAGE1']
